# Causal decomposition of Black-white disparities in NIH’s selection of proposals for panel discussion

**DOI:** 10.1371/journal.pone.0358838

**Published:** 2026-10-28

**Authors:** Shreya Prakash, Carole J. Lee, Elena A. Erosheva

**Affiliations:** 1 Department of Statistics, University of Washington, Seattle, Washington, United States of America; 2 Department of Philosophy, University of Washington, Seattle, Washington, United States of America; 3 Center for an Informed Public, University of Washington, Seattle, Washington, United States of America; 4 School of Social Work, University of Washington, Seattle, Washington, United States of America; 5 Center for Statistics and the Social Sciences, University of Washington, Seattle, Washington, United States of America; Visva-Bharati University: Visva-Bharati, INDIA

## Abstract

Prior research on NIH R01 grants peer review found that selection of proposals for panel discussion constituted the decision point with the largest contribution to Black-white NIH funding disparities. We use a causal decomposition framework to understand what factors causally contribute to reducing Black–white disparities in selection into discussion, and to assess how recent procedural changes may affect Black–white disparities in selection into discussion. We first use causal decomposition methodology to explore whether hypothetical interventions eliminating Black-white disparities and/or differences in attributes would reduce Black-white disparities in selection of proposals for panel discussion. The attributes we consider include those associated with applicants (degree, career stage, their institution’s funding bin) and their submitted proposals (application type, amended status, Preliminary Overall Impact Score, and NIH assigned Administering Organization and Integrated Review Group). Under reasonable assumptions, we find that among the attributes considered, Preliminary Overall Impact Score was the only one that, after equalizing, could dissolve Black-white disparities in selection into discussion. In addition, we conduct a thought experiment to explore potential impacts of the NIH’s move to binary scoring for the Investigator and Environment criteria in the new Simplified Peer Review Framework on Black-white disparities in selection into discussion. Analyses from our thought experiment suggest that changing to binary scoring of the Investigator-and-Environment factor may not have any effect on disparities in selection into discussion. Overall, under the assumptions of the causal decomposition framework, these results suggest that efforts aimed at reducing disparities in selection into discussion should focus on identifying upstream, policy-relevant, and practically feasible interventions for eliminating racial disparities in Preliminary Overall Impact Scores. In contrast, the transition to the Simplified Peer Review Framework is unlikely to substantially reduce disparities in selection into discussion.

## Introduction

The National Institutes of Health (NIH) “is the largest public funder of biomedical research in the world” [1]. The NIH funds research through a competitive peer review process. A major mechanism for this funding is the Research Project Grant (R01), which supports investigator-initiated research aligned with the mission of the NIH and its Institutes and Centers [2]. The R01 grant “is the original and historically oldest grant mechanism used by NIH” [3].

Research has demonstrated persistent Black-white disparities in funding at the NIH, even as of 2022 [4], more than ten years after Ginther et al’s first report (2011). Portions of the funding gap have been attributed to different structural inequities in science. Ginther and colleagues [5] found that, in submitted Biosketches, new and experienced Black Principal Investigators (PIs) publish in journals with lower impact factors, have fewer field-normalized citations, and report fewer publications than their white peers–-and that these bibliometric differences account for roughly half the funding gap. Lauer et al [6] discovered that part of the funding disparity could be attributed to the lower award rates at Centers and Institutes to which Black PI’s disproportionately apply [6]–-and that this fully accounts for previously observed associations between PI race and proposal topic choice [7]. Inequities in mentoring and co-author networks are also thought to play a role: Warner and colleagues found that within the context of Harvard Medical School, an NIH applicant’s network reach–-measured as the intra-institutional number of the applicant’s co-authors plus the intra-institutional number of their co-authors’ co-authors–-was associated with receiving an NIH R01 grant [8]. Although the Black-white funding gap at NIH has narrowed in recent years, it has not been eliminated (Refer to Figure 8 of [9]). The persistence of Black–white disparities raises concerns about equity in access to research funding and the potential loss of scientific contributions from underrepresented groups. The continued attention to this issue in recent work following Ginther et al. (2011) underscores its ongoing importance (e.g.[6,10],). However, most of this work relies on associational analyses and does not establish which factors causally contribute to disparities or which changes to the review process would meaningfully reduce them.

In this paper, our objective is to use a causal decomposition framework to understand what factors causally contribute to reducing Black–white disparities in selection into discussion, and to assess how recent procedural changes may affect Black–white disparities in selection into discussion.

Research grant proposal applications must successfully surmount multiple hurdles to secure NIH R01 funding, as shown in Fig 1, with the first being selection into discussion by Scientific Review Groups (SRGs). NIH SRGs–-study sections composed of subject-area experts who conduct the peer review of R01 applications–-are organized within broader Integrated Review Groups (IRGs), which cluster related study sections by scientific discipline.

**Fig 1:**
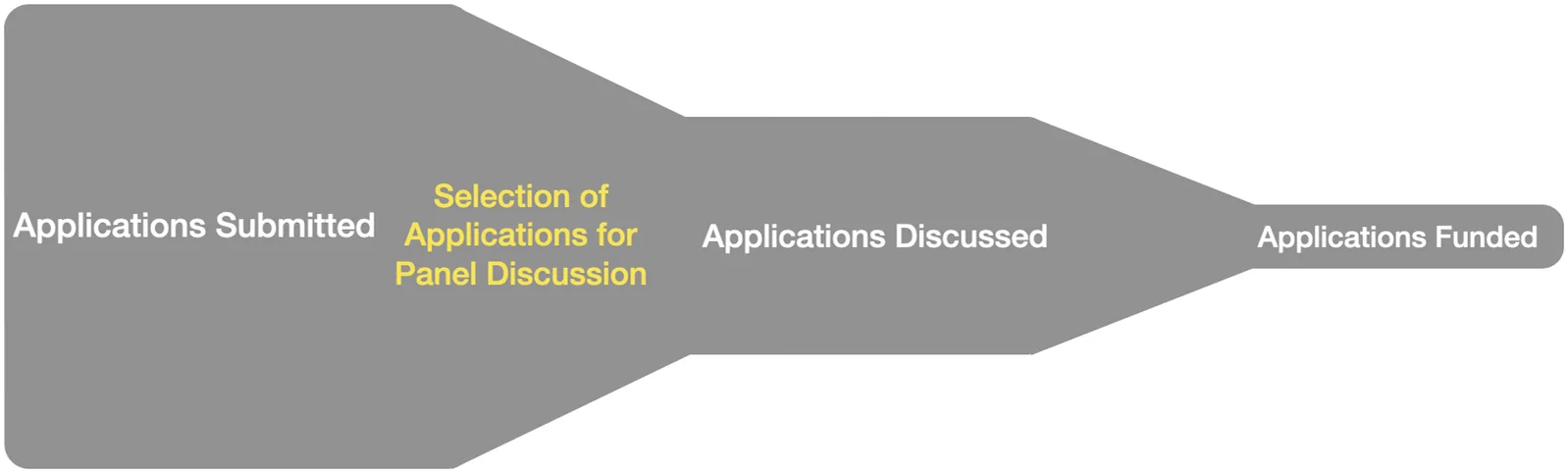
Overview of the NIH R01 peer review process. Figure widths reflect the approximate proportion of applications at each stage: all applications receive preliminary scores; roughly half are selected for discussion; and only a smaller subset ultimately receive funding. White text denotes the three major stages. Yellow text highlights a key review process–-the selection of proposals for panel discussion. Adapted from Fig. 1 in Hoppe et al. [7].

In the Enhanced Peer Review Process used by the NIH between January 25, 2009 and January 24, 2025 [11], assigned reviewers evaluated the proposed research and the biosketch, which contains applicants’ names, institutional affiliations, and selected publications [7,12], and provided preliminary (i.e., before panel discussion) criterion scores for Significance, Investigators, Innovation, Approach, and Environment as well as Preliminary Overall Impact Scores for each submitted application (see Table 1 for descriptions). Each preliminary criterion was scored on a scale from 1 to 9, with 1 being the best and 9 the worst. Preliminary Overall Impact Scores were used to nominate which applications–-typically half based on Preliminary Overall Impact Scores–-were not to be discussed. This process of nominating applications to not be discussed based on a Preliminary Overall Impact Score threshold is known as ‘streamlining’ [13]. Applications that were not ‘streamlined’ would go on to be discussed.

**Table 1:** NIH’s descriptions of the five review criteria under the Enhanced Peer Review Process. ‘Reviewers will provide’ a preliminary ‘overall impact/priority score to reflect their assessment of the likelihood for the project to exert a sustained, powerful influence on the research field(s) involved, in consideration of the following five core review criteria, and additional review criteria (as applicable for the project proposed)’ [11,14].

Enhanced Peer Review Process (for due dates before Jan 25, 2025)	
*All five scored criteria are considered in the Preliminary Overall Impact Score*	
Criterion (Scored 1–9)	Description
**Significance**	Does the project address an important problem or a critical barrier to progress in the field? If the aims of the project are achieved, how will scientific knowledge, technical capability, and/or clinical practice be improved? How will successful completion of the aims change the concepts, methods, technologies, treatments, services, or preventative interventions that drive this field?
**Innovation**	Does the application challenge and seek to shift current research or clinical practice paradigms by utilizing novel theoretical concepts, approaches or methodologies, instrumentation, or interventions? Are the concepts, approaches or methodologies, instrumentation, or interventions novel to one field of research or novel in a broad sense? Is a refinement, improvement, or new application of theoretical concepts, approaches or methodologies, instrumentation, or interventions proposed?
**Approach**	Are the overall strategy, methodology, and analyses well-reasoned and appropriate to accomplish the specific aims of the project? Are potential problems, alternative strategies, and benchmarks for success presented? If the project is in the early stages of development, will the strategy establish feasibility and will particularly risky aspects be managed? If the project involves clinical research, are the plans for (1) protection of human subjects from research risks, and (2) inclusion of minorities and members of both sexes/genders, as well as the inclusion of children, justified in terms of the scientific goals and research strategy proposed?
**Investigator(s)**	Are the PD/PIs, collaborators, and other researchers well suited to the project? If Early Stage Investigators or New Investigators, do they have appropriate experience and training? If established, have they demonstrated an ongoing record of accomplishments that have advanced their field(s)? If the project is collaborative or multi-PD/PI, do the investigators have complementary and integrated expertise? Are their leadership approach, governance, and organizational structure appropriate for the project?
**Environment**	Will the scientific environment in which the work will be done contribute to the probability of success? Are the institutional support, equipment, and other physical resources available to the investigators adequate for the project proposed? Will the project benefit from unique features of the scientific environment, subject populations, or collaborative arrangements?

Selection for panel discussion included an additional step. That is, after ‘streamlining’, panel reviewers assessed applications nominated not to be discussed in a randomized order to decide if any should be added to the discussion [13]. For each proposal nominated not to be discussed, all reviewers without a conflict of interest must have agreed unanimously not to discuss that application; otherwise, applications would have been moved along to the discussion phase [13]. All applications selected for SRG panel discussions were provided final Overall Impact Scores. At the funding step, these final Overall Impact Scores guided the NIH’s Centers’ and Institutes’ funding decisions along with a variety of other factors such as pubic health burden, programmatic priorities, overall portfolio balance and budgets at the funding institutes [7,12].

On January 25th, 2025, the NIH introduced the new Simplified Peer Review Framework. The goals of the Simplified Peer Review Framework are to 1) simplify the peer review process to be more focused on the key questions necessary to assess scientific and technical merit, 2) reduce reviewer burden, and 3) mitigate the effect of reputation bias [15]. Under this new system, instead of scoring five criteria individually, reviewers score three broad factors that encompass slightly updated versions of the previous review criteria [16], as shown in Table 2. A key simplification aimed at helping “put applicants on equal footing” involves combining the Investigator and Environment criteria into a single binary assessment to be scored as “gaps identified” or not [17]. A major goal of this specific simplification is to “mitigate the effect of reputational bias” [17], a plausible contributor to Black-white disparities [5].

**Table 2:** NIH’s descriptions of the Review Criteria Under the Simplified Peer Review Framework [15,16]. The Simplified Peer Review Framework reorganizes NIH’s five individual review criteria (Table 1) into three broader factors: (1) Importance of the Research, (2) Rigor and Feasibility, and (3) Expertise and Resources. The most notable changes are: (i) Significance and Innovation are combined into a single 1–9 score under Factor 1, and (ii) Investigator and Environment are merged into a single binary score under Factor 3. These changes are detailed in NIH guidance and webinars [15,16].

Simplified Peer Review Framework (for due dates on/after Jan 25, 2025)	
*All three factors are considered in the Preliminary Overall Impact Score*	
**Factor (Score)**	**Evaluation Criteria**
**Factor 1: Importance of the Research (Scored 1–9)**	**Significance:** Evaluate the importance of the proposed research in the context of current scientific challenges and opportunities. Does the application address a significant knowledge gap, critical problem, or valuable conceptual/technical advance? Assess the rigor and justification of the scientific background. **Innovation:** Evaluate the influence of innovation on the importance of the proposed research. Does the project apply novel concepts, methods, or technologies, or use existing approaches in novel ways? Projects without novelty may still be highly important to the field.
**Factor 2: Rigor and Feasibility (Scored 1–9)**	**Approach:** Evaluate the scientific quality of the work. Assess whether it is likely to yield compelling, reproducible results and whether the proposed studies are feasible within the proposed timeline. **Rigor:** Evaluate experimental design, controls, sample size, plans for analysis, and whether biological variables (e.g., sex or age) are addressed. For human or animal studies, assess the rigor of interventions, outcome justification, generalizability, and population appropriateness. **Feasibility:** Evaluate whether the research plan is realistic, including recruitment and retention strategies (for human studies), study milestones, and likelihood of achieving goals across diverse subgroups.
**Factor 3: Expertise and Resources (Binary: Appropriate / Gaps Identified)**	**Investigators:** Are the PD/PIs and collaborators well qualified? Do they have relevant experience, training, and complementary expertise? **Environment:** Does the institutional setting, available resources, and infrastructure support project success? Proposals flagged as having “gaps identified” require an explanation. This factor is not numerically scored but must be assessed for appropriateness.

In this paper, we undertake causal analyses to evaluate which interventions could potentially decrease Black-white disparities in selection of proposals for panel discussion. We focus on this stage for two main reasons. First, this decision point culls the largest number of proposals. Second, this step in the process was shown to constitute “the largest single contribution” to Black-white disparities in NIH grant review and funding [7].

Despite the importance of this decision point in the review process, it has not been investigated from a causal analysis perspective. Most analyses aimed at understanding Black-white disparities in funding, including some of the most groundbreaking work [5–7,10], have reported associations and, as such, refrained from drawing causal conclusions. For example, the only study that investigated selection of proposals for panel discussion [7] found significant associations between PI race, application resubmission, institutional prestige, and career stage and the outcome of selection of proposals for panel discussion; however, these associations estimated from observational data were not evaluated for their causal contribution to disparities in selection into discussion. Experimental efforts to identify causal mechanisms for racial disparities in peer review outcomes include a study that anonymized PI identities [12] and another study that swapped social identities of the names listed on otherwise identical proposals [18]. However, these studies focused primarily on the causal role of perceived applicant race and did not consider other factors that could impact disparities in scoring and funding. Identifying hypothetical interventions that could reduce or eliminate racial disparities in selection into discussion could inform where policies could be directed most effectively.

In the preliminary analysis section, we start by confirming Hoppe et al.’s [7] finding of substantial Black-white disparity in selection into discussion with our data and illustrate variation in these disparities across NIH’s Integrated and Scientific Review Groups. Then, to gain insight into the scoring and selection process, we extend the causal decomposition framework introduced by Vanderweele et al. [19] for the peer review context. The causal decomposition overview section outlines causal decomposition methodology, its assumptions, and its adaptation to the peer review context. Importantly, the causal decomposition framework does not estimate the causal effect of race. Rather, it begins by estimating the observed Black–white disparity in selection into discussion and then asks how this disparity would change under hypothetical interventions of certain attributes [19]. In the first part of the results section, we use this framework to quantify: (i) the observed Black–white disparity in selection into discussion; (ii) how much of this disparity would be reduced if the distribution of a given attribute (e.g., PI career stage or institutional prestige) were equalized across groups; and (iii) how much of the disparity would remain after such equalization.

In the second part of the results section, we examine counterfactual questions about how procedural changes akin to the Simplified Peer Review Framework’s move to replace the Investigator and Environment 1–9 scores with one binary Factor 3 score might affect disparities in selection for discussion. We single out this procedural change in our analysis as it represents an intervention introduced by the NIH with the stated goal of “mitigat[ing] the effect of reputational bias” [17], a factor that may contribute to Black–white disparities in peer review [5]. While we do not have access to data from NIH’s new Simplified Peer Review, we can still consider a thought experiment in which we use available data from the NIH’s Enhanced Peer Review to binarize the Investigator and Environment criteria separately since, after deciding on each Investigator and Environment criteria score, one can relate these two binarized scores to the combined Factor 3 score as outlined in Fig 2. To implement our thought experiment, we use two conceptual extremes regarding how criterion scores relate to Preliminary Overall Impact Score since that mechanism is unknown.

**Fig 2:**
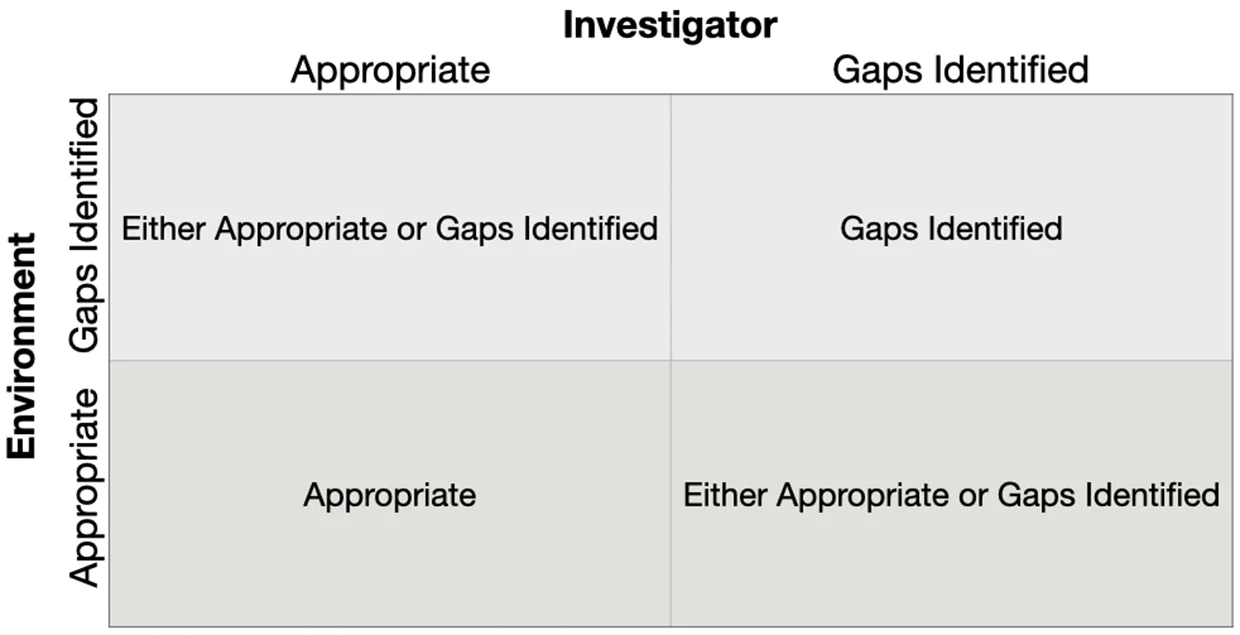
Relationship between the binary Investigator and Environment criteria and their combined score. Individual Investigator and Environment scores are shown in the margins, and the combined binary Investigator and Environment score is shown within the cells.

As with any statistical analysis, our causal decomposition analysis relies on assumptions, most of which are not directly testable from the observed data. Thus, the causal interpretations of our results should be understood as conditional on the assumptions underlying the causal decomposition framework. In the causal decomposition overview section, we describe these assumptions in technical detail and discuss their plausibility in the context of NIH peer review.

Under the assumptions outlined in the causal decomposition overview section, our causal decomposition analysis yields several causal conclusions. First, eliminating racial disparities and/or differences in one of the attributes, in particular Preliminary Overall Impact Score, can dissolve the disparity in selection into discussion. This result was not guaranteed a priori because, as we discuss in the paper and S1 Text, selection into discussion is not deterministically driven by Preliminary Overall Impact Scores. Second, we find that the impact of changes in the Simplified Peer Review Framework on disparities in selection into discussion depends on how reviewers evaluate the binary criteria as well as on the underlying causal relationship between the criterion scores and Preliminary Overall Impact Scores. The results from our thought experiment show that two conceptual extremes for this causal relationship can theoretically lead to different effects that some may find surprising (see Figs 7 and S5). However, if the criteria are evaluated using cutoffs that reflect historical discussion rates at the NIH, our thought experiment suggests that neither extreme would substantially alter Black-white disparities in selection into discussion. Thus, under the assumptions of the causal decomposition framework, we conclude that while reductions in racial disparities in Preliminary Overall Impact Scores may impact disparities in selection into discussion, the transition to the Simplified Peer Review Framework is unlikely to do so.

### Preliminary analysis

Our dataset consists of preliminary peer review scores and several PI and application characteristics for a subset of R01 submissions by Black and white PIs from 2014–2016 [10], and is publicly available on the Open Science Framework [20] (https://osf.io/4d6rx/). The dataset contains all submissions from Black PIs and a random sample of submissions from white PIs. 22% of the PIs in the data are identified as Black, while 78% are white. Additional details on our dataset and its construction are provided in Section S3.1 in S3 Text.

We observe that 54% of proposals are selected for discussion, aligning with the NIH’s standard that approximately half of proposals are discussed; however, there is a great deal of variability across Integrated Review Groups (IRGs) [21]. We note that 56.4% (Cred. Interval = [54.4%, 58.2%]) of white PIs are selected for panel discussion, while only 43.9% (Cred. Interval = [40.6%, 47.3%]) of Black PIs are selected. Because we observe all Black PI applications, whereas the white PI data comprise both a matched subset of all white PIs and a randomly sampled subset (see [10] for details on the publicly available dataset), the credible intervals are conservative and not directly comparable across groups. Overall, there is a substantial 12.3% (Cred. Interval = [11.3%, 16.3%]) disparity in selection for discussion toward Black PIs. This is similar to the findings of Hoppe et al. (2019), who observed a 13.4% disparity in their analysis of grant applications submitted between 2011 and 2015 [7]. Fig 3 presents discussion rates and Black-white disparities in discussion rates by IRG. We observe considerable variability, with discussion rates across IRGs ranging from 40% to 57%. Discussion rates further vary by SRG; Fig 3 (bottom panel) shows SRG discussion rates for SRGs within the largest IRG range from 41% to 71%.

**Fig 3:**
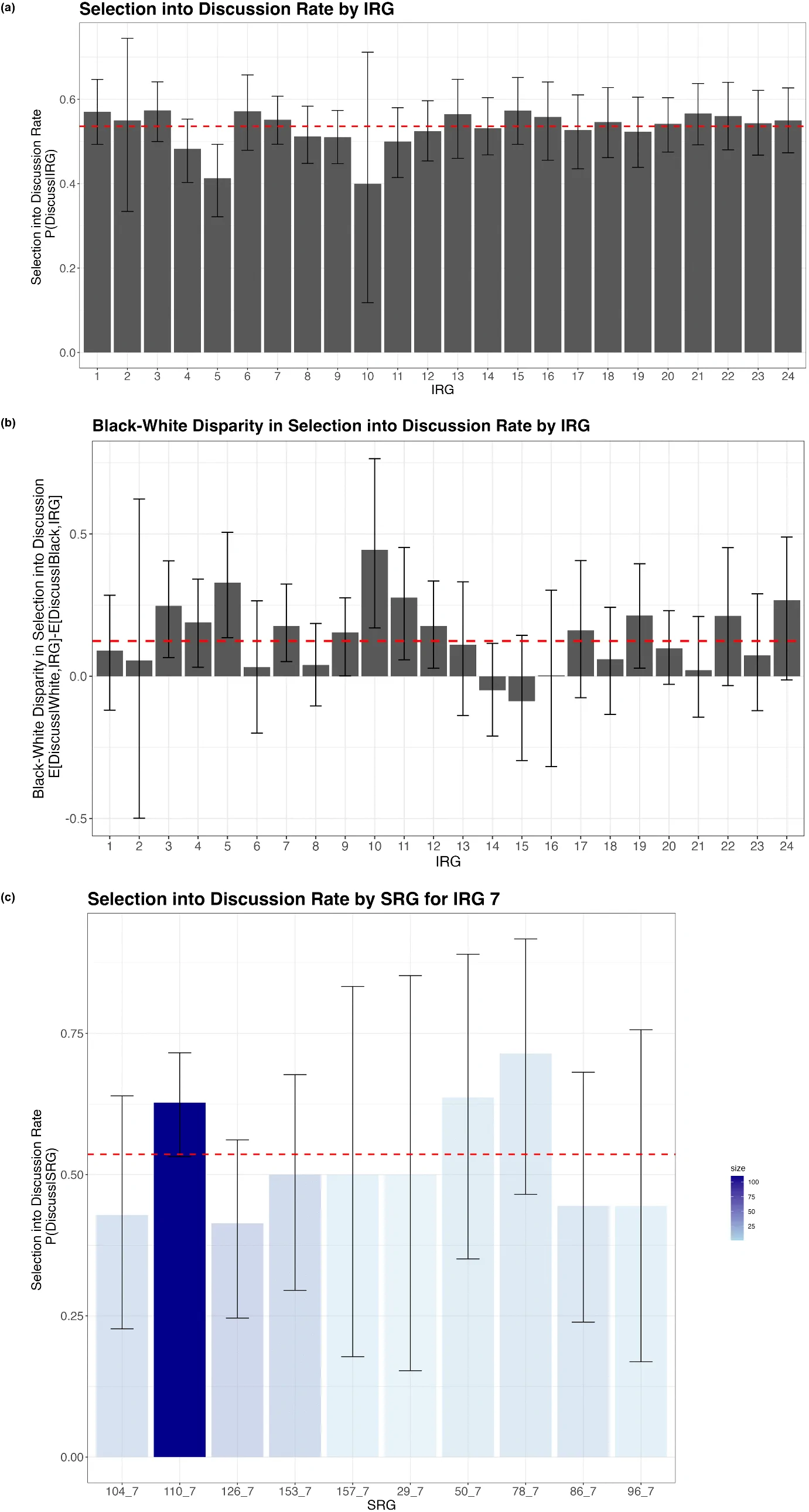
Discussion rates and Black–White disparities across IRGs and SRGs. (a) Discussion rates. (b) Black-white disparities in selecting into discussion by IRG. (c) Discussion rates and the associated 95% Credible Intervals for SRGs within the largest IRG (IRG 7). In all panels, the red dashed line is the overall discussion rate of 0.54 (0.51, 0.56) and the overall disparity of −0.12 (−0.16, −0.08). Credible Intervals were obtained using a Bayesian bootstrap with 500 posterior samples.

Discussion rates vary substantially based on Preliminary Overall Impact Scores, as well as Investigator and Environment criterion scores (see S1 Table). Specifically, almost no proposals with preliminary criteria scores above 5 are discussed, while the vast majority of proposals with Preliminary Overall Impact Scores of 1 (the highest score) are discussed. However, both NIH documentation and our data suggest that while selection for panel discussion is primarily driven by Preliminary Overall Impact Score, the process is not entirely deterministic. As outlined in S2 Text, our analysis demonstrates that while these scores play a central role, there may be other additional factors that influence which applications are ultimately selected for discussion.

### Causal decomposition overview

This overview summarizes the causal decomposition methodology introduced by VanderWeele and Robinson [19] and describes how we extend it in the context of NIH peer review. Full methodological details – including identification results for each hypothetical intervention, the Bayesian estimation procedure, model specification, model evaluation, as well as additional technical derivations and implementation details – are provided in S3 Text.

We begin by constructing a causal diagram (Fig 4) informed by domain knowledge of the NIH peer review process [21]. In this diagram, the presence of a directed edge between two variables indicates the possibility of a causal relationship, while the absence of an edge reflects an assumed lack of causal influence.

**Fig 4:**
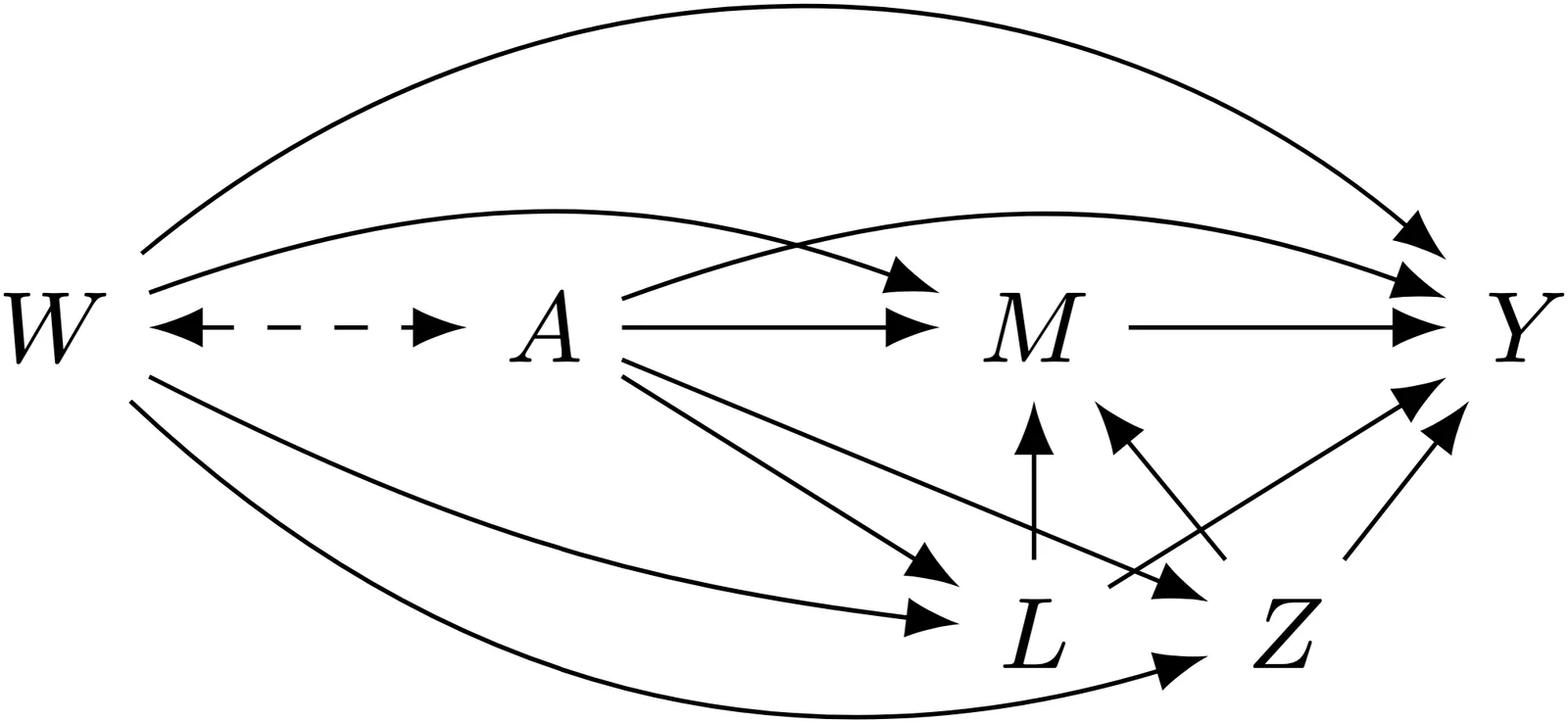
Causal Diagram of NIH Peer Review Process. This diagram illustrates the causal relationships within the NIH peer review process. In this representation, *A* corresponds to the PI race, with *A* = 0 representing white PIs and *A* = 1 representing Black PIs. The outcome, *Y*, signifies the selection of proposals for discussion. *W* denotes the PI’s ethnicity and gender, regarded as protected attributes not currently under examination. We allow for an association between ethnicity, gender (*W*), and PI race (*A*), as indicated by the dotted bi-directional arrow. Intermediate variables linking PI race to selection into discussion include: *M*, the Preliminary Overall score, *Z*, the Application Type, Amended Status, Career Stage, Degree Category, and Funding Bin jointly, and *L*, the Integrated Review Group (IRG), Scientific Review Group (SRG), and Administering Organization.

The variable *A* corresponds to the PI race, with *A* = 0 representing white PIs and *A* = 1 representing Black PIs. The outcome, *Y*, signifies the selection of proposals for panel discussion. *W* denotes the PI’s ethnicity and gender, regarded as protected attributes not currently under examination. We allow for an association between ethnicity, gender (*W*), and PI race (*A*), as indicated by the dotted bi-directional arrow. Intermediate variables linking PI race to selection into discussion include: *M*, the Preliminary Overall Impact Score, *Z*, the Application Type, Amended Status, Career Stage, Degree Category, and Funding Bin jointly, and *L*, the Integrated Review Group (IRG), Scientific Review Group (SRG), and Administering Organization. This causal diagram reflects our understanding of the NIH review process and serves as the foundation for our causal analysis. An alternative causal diagram that accounts for self-selection is presented in S4 Fig.

We extend the causal decomposition framework introduced by VanderWeele et al. [19] to develop mathematical tools that could allow one to evaluate how changes in the distribution of specific attributes–-such as Preliminary Overall Impact Scores or degree category–-contribute to Black–white disparities in selection into discussion. VanderWeele et al. [19] propose this framework to “provide a causal interpretation of race coefficients... without defining potential outcomes for race itself,” noting that “there are... no reasonable hypothetical interventions on race.” Instead, they ask “how much a racial inequality could be eliminated by intervening on a different variable,” shifting the focus to factors that are more manipulable than race itself [19].

While it may not be feasible to directly change distributions of these other attributes through experiments – as attributes like PI degree category are not easily manipulated through experimental interventions – we can still consider hypothetical interventions that change these distributions as a way to gain insight. Such analyses help us understand how disparities might change under policies that influence these factors. For example, we might consider a hypothetical intervention that equalizes Preliminary Overall Impact Scores between Black and white PIs (i.e., removes any Black-white disparities in Preliminary Overall Impact Scores). Analyzing such a scenario can help us understand how much of the disparity in selection for discussion is driven by the score differences, and guide policies that would target underlying factors.

We define three key estimands to assess disparities in selection into discussion between Black and white PIs. The **observed disparity** (OD; Eq 1) captures the difference in discussion rates between Black and white PIs. The **disparity reduced** (DRed; Eq 2) is the change in racial disparities resulting from a hypothetical intervention to equalize the distribution of a given attribute–-such as Preliminary Overall Impact Scores–-between Black and white PIs. To formalize this, we let $M^{*}$ denote a value drawn from the conditional distribution of Preliminary Overall Impact Scores for white PIs, given gender, ethnicity, IRG, SRG, and Administering Organization: P(M∣A=0,W,L). Using the potential outcomes framework [22], which allows us to define outcomes under hypothetical scenarios, we define $Y(M^{*})$ as an indicator for whether a proposal would be selected for discussion after the hypothetical intervention to equalize the distribution of Preliminary Overall Impact Scores between white and Black PIs. The **Disparity Remaining** (DRem; Eq 3) reflects the part of the disparity that remains after such an intervention. To mirror NIH’s streamlining practice, all potential outcomes (e.g., $Y(M^{*})$) are defined under the constraint that the discussion rate within each SRG *k* is fixed at its observed value, $d_{k}$.


$$\begin{array}{c} \text{Observed Disparity: }E[Y|A=0]-E[Y|A=1] \end{array}$$ (1)

$$\begin{array}{c} \text{Disparity Reduction: }E[Y(M^{*})|A=1]-E[Y|A=1]\;s.t.\;E[Y_{k}(M^{*})]=d_{k} \end{array}$$ (2)

$$\begin{array}{c} \text{Disparity Remaining: }E[Y|A=0]-E[Y(M^{*})|A=1]\;s.t.\;E[Y_{k}(M^{*})]=d_{k} \end{array}$$ (3)

We also study a specific hypothetical intervention motivated by NIH’s Simplified Peer Review Framework: binarizing the Investigator and Environment criterion scores. To assess its impact, we define the **thresholded disparity** (TD; Eq 4), which captures the difference in selection into discussion between Black and white PIs after intervening to binarize the Investigator and Environment criterion scores.


$$\begin{array}{c} \text{Thresholded Disparity: }E[Y(M_{i,e}^{t})|A=0]-E[Y(M_{i,e}^{t})|A=1]\;s.t.\;E[Y_{k}(M_{i,e}^{t})]=d_{k} \end{array}$$ (4)

Formally, let $M_{i,e}^{t}$ denote the binarized Investigator and Environment criterion scores (as a stacked vector), based on a predefined threshold *t*. If $M_{i,e}\leq t$, then $M_{i,e}^{t}=[1,1]$ (indicating a strong score); o*t*herwise, $M_{i,e}^{t}=[5,5]$ (indicating a poor score). The potential outcome $Y(M_{i,e}^{t})$ represents whether a proposal would be selected for discussion under this binarized scoring.

The following assumptions are sufficient to nonparametrically identify the Disparity Reduction, Disparity Remaining, and thresholded disparity using observational data. Formal identification proofs, along with a discussion of how self-selection affects the definition and identification of these estimands, are provided in the S5 and S6 Texts.

1. Partial Interference: Interference only occurs within each SRG
2. Consistency at the SRG level: If $M_{k}=m$, then $Y_{k}=Y_{k}(m)$
3. Effect of *M* on the outcome *Y* is unconfounded at the SRG level
4. Positivity assumptions at the SRG level

Due to the streamlining procedure, an intervention on the score of one proposal can affect how other proposals are selected to be discussed within the same SRG, resulting in interference (see S3 Fig for simplified interference structure). However, this interference is limited to within each SRG, making the partial interference assumption reasonable. The consistency assumption is also reasonable in this setting: if a proposal receives a particular score, then the observed selection into discussion outcome reflects what would happen under that score. The no unmeasured confounding assumption requires that, conditional on observed covariates (i.e., gender, ethnicity, IRG, SRG, and Administering Organization), there are no unobserved variables that influence both the intervened-on attribute (i.e., Preliminary Overall Impact Score) and selection into discussion. A key concern for this assumption is the presence of unobserved aspects of proposal quality or evaluation context that may affect both preliminary scores and discussion decisions. Many dimensions of proposal quality are reflected in the criterion scores and Preliminary Overall Impact Score themselves; however, it is possible that some aspects of proposal quality or evaluation context are not fully captured by these scores and remain unobserved. To mitigate confounding, we adjust for covariates selected based on the causal diagram (Fig 4), including structural, application, and investigator characteristics. In particular, conditioning on IRG, SRG, and Administering Organization accounts for systematic differences in reviewer composition and evaluation standards, while application and investigator characteristics capture key observable dimensions of proposal quality and/or review context. While these adjustments address major sources of observed variation, the no unmeasured confounding assumption cannot be verified and may be violated if important aspects of proposal quality or evaluation context are not fully captured. The causal diagram (Fig 4) clarifies the assumptions under which identification holds, and our results should be interpreted in light of this assumed causal structure. Lastly, the positivity assumption is also plausible, since our data includes both Black and white PIs across all relevant subgroups.

The set of covariates used for adjustment is determined by the directed acyclic graph and corresponding identification forms. These covariates are included to account for differences in observed context that may influence both the attribute being intervened on and the outcome. Conditioning on these variables allows us to compare outcomes between Black and white PIs who are similar along these observed dimensions. For example, when evaluating interventions on preliminary impact scores, we adjust for PI characteristics (e.g., career stage, degree category, and prior funding), proposal characteristics (e.g., application type and amendment status), and structural features of the review process (e.g., IRG and administering organization), with SRG included as a random effect. Full details of the covariate sets corresponding to each intervention are provided in Tables S5 and S6.

To estimate Disparity Reduction, Disparity Remaining, and thresholded disparity, we develop a Bayesian estimation approach. This approach allows for interventions on multiple attributes, incorporates NIH’s ~50% discussion rate constraint, accounts for the hierarchical peer review structure, and provides uncertainty quantification. It estimates posterior distributions for each estimand and yields corresponding credible intervals. Using simulations under a known data-generating process, we assess bias of the proposed approach and evaluate the potential impact of interference on estimation. The simulation design and results are provided in S4 Text and S7 Table.

## Results

### Causal decomposition analysis of hypothetical interventions equalizing Black-white differences in attributes

In this section, we focus on several variables that may influence a proposal’s chances of being discussed: the Preliminary Overall Impact Score, application type, amended status, degree category, funding bin, career stage, Administering Organization, and IRG, which is a proxy for scientific area. Given that distributions of these variables differ substantially by race, we investigate – via causal decomposition analyses – whether hypothetical interventions of eliminating these disparities and/or differences would influence racial disparities in selection into discussion.

Observed disparities and/or differences in the investigator and application characteristics by race are substantial. The observed distribution of mean Preliminary Overall Impact Scores differs between Black and white PIs, with white PIs tending to receive lower (better) scores. We also observe notable racial differences in other key attributes: white PIs have a 5.1% higher rate of renewed applications, 7.9% more hold a Ph.D., while 4.3% and 3.4% more Black PIs hold an M.D. or other degrees, respectively. Additionally, 9.2% more Black PIs are Early Stage Investigators, 6.5% more are non-Early-Stage-Investigators, and 15.7% more white PIs are Experienced Investigators. Lastly, the distributions of Administering Organization and IRGs also differ between Black and white PIs. We also observe differences in the PI’s institution’s funding bin which is a proxy for its prestige, where lower bins indicate higher prestige [10]. These differences highlight potential contributors to disparities in selection for discussion and motivate the use of hypothetical interventions to assess whether targeting these factors could reduce those disparities. Tables and figures in the Supporting Information (S1 Text, S1-S3 Tables, and S1-S2 Figs) provide further details on these comparisons.

We examine several hypothetical interventions eliminating racial disparities and/or differences in: (1) mean Preliminary Overall Impact Score, (2) application type and amended status jointly, (3) degree category, funding bin, and career stage jointly, and (4) Administering Organization and IRG jointly. These interventions are motivated by observed racial differences in the distributions of these attributes and are grounded in previous research that examined success in securing NIH funding [5–7,10]. Ginther et. al found that those with strong priority scores (scores given after SRG panel discussion), were equally likely to receive funding, irrespective of the PI’s race [5]. Hoppe et al. found significant marginal effects for (1) application resubmission, with new applications being significantly less likely to be discussed and awarded than resubmitted applications; (2) institution prestige, with applications from less prestigious institutions (those that receive less funding) being significantly less likely to be discussed and awarded; (3) career stage, with early career investigators being significantly more likely to be discussed and awarded; and (4) topic choice, suggesting that preferences for topics “less likely to excite the enthusiasm of the scientific community” could lead to lower discussion and funding rates, potentially creating a vicious cycle [7]. Subsequently, Lauer et al. refuted Hoppe et al.’s claim that topic choice is the primary reason for the funding gap and instead suggested that differences in funding rates between Institutes and Centers to which PI applications are assigned play a larger role [6].

We estimate the expected Disparity Reduction and Disparity Remaining in selection into discussion under hypothetical interventions aimed to equalize the attribute(s) of interest among Black and white PIs [19,23]. To do this, we randomly assign the attribute(s) under consideration among Black PIs such that they follow the same distribution as white PIs conditional on gender, ethnicity, IRG, SRG, and Administering Organization. (If the intervention under consideration is for IRG and Administering Organization jointly, we do not include IRG, SRG, and Administering Organization among conditioning variables). To estimate the disparity reduced, we use a causal decomposition analysis, assuming the causal structure in Fig 4 and accounting for SRG-level discussion rates, so that comparisons are made within an SRG-constrained competitive selection process. We use logistic regression to model the selection process. We use either linear or binary/multinomial logistic regressions, as appropriate, to model the attribute(s) under consideration. For the joint interventions, we first model the distribution of one of the attributes and then model the conditional distribution of the other attributes given the previously modeled attributes. Summaries of the interventions, estimands, identification assumptions, and identification forms for all interventions considered in the equalization analysis are provided in S5 Table.

Under the assumptions of the causal decomposition framework, our findings, summarized in Fig 5, indicate that equalizing mean Preliminary Overall Impact Scores would dissolve racial disparities (Disparity Remaining Estimate = 0.1%, 95% Cred. Interval = [−2.7%, 2.9%]); however, equalizing distributions of application type, amended status, degree category, funding bin, career stage, IRG, and/or Administering Organization by race results in minimal reductions in the disparity in selection into discussion.

**Fig 5:**
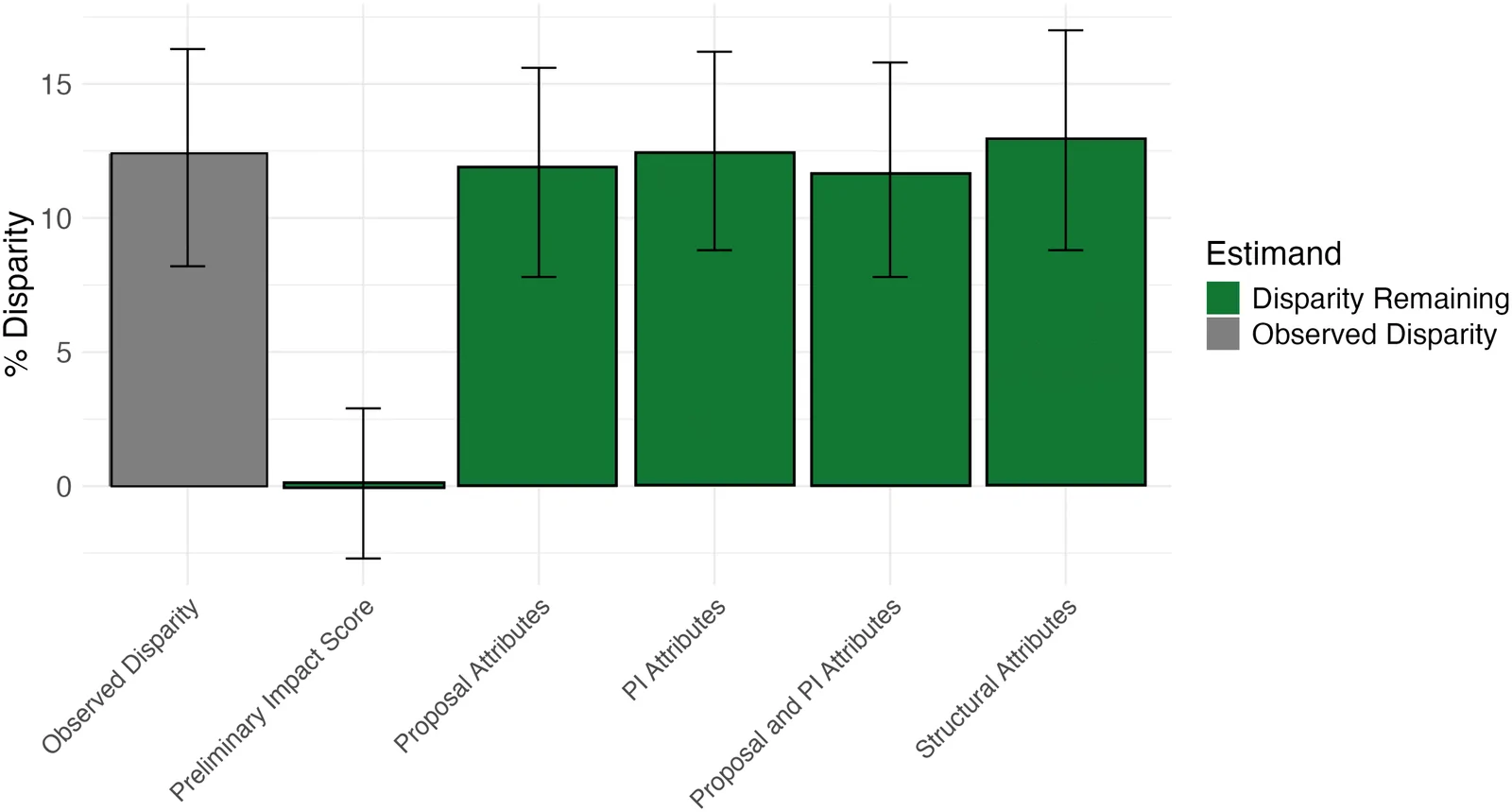
Estimated Overall Disparity and Disparity Remaining. Estimated Disparity Remaining for Preliminary Overall Impact Scores, proposal attributes (application type and amended status), PI attributes (career stage, degree category, and institutional funding bin – a proxy for institution prestige), and structural attributes (IRG and Administering Organization). Positive values indicate disparity against Black PIs. Included 95% Credible Intervals. Estimates and intervals were obtained using Bayesian estimation.

### Thought experiment on binary investigator and environment scoring under the simplified peer review framework

On January 25th, 2025, NIH implemented the Simplified Peer Review Framework [16]. Tables 1 and 2 demonstrate the key differences between the Enhanced Peer Review Process and Simplified Peer Review Framework. A key simplification is combining the evaluation of the Investigator and Environment into a single binary score, labeled as Factor 3, which will be rated as either “appropriate” or “gaps identified”. This “will help put applicants on equal footing”, aiming to “mitigate the effect of reputational bias” [17], a plausible contributor to Black-white disparities [5]. Although we cannot fully assess changes due to combining the Investigator and Environment into a single binary score, we can use our causal decomposition framework to assess effects of binarization of both Investigator and Environment criterion scores on racial disparities in selection into discussion. Assuming that reviewers determine binary appropriateness based on the previous score being below a certain threshold, we consider a thought experiment by binarizing the Investigator and Environment scores separately at those thresholds while keeping all other criterion scores as they are. We do not know exactly where the psychological threshold for “appropriate” versus “gaps identified” will fall relative to the previous 9 point scale, so we perform a range of analyses with different thresholds. If the threshold is very high (recall that higher scores on the 9 point scale are worse), almost all proposals will be marked as “appropriate”, essentially leading to parity. Similarly, if the threshold is very low, almost all proposals will be marked as “gaps identified”, also resulting in parity. In what follows, we refer to the Investigator and Environment criterion scores as applicant-related scores, and the Significance, Innovation, and Approach criterion scores as proposal-related scores.

We propose a thought experiment where reviewers map their numerical scores (1–9) for rating Investigator and Environment criteria onto binary “appropriate” or “gaps identified” decisions. Each of the three reviewers would give one of these two designations. To model the impact onto discussion, we assume that “appropriate” would correspond to a numeric Investigator/Environment score that indicates the proposal will likely proceed to be discussed. Likewise, “gaps identified” would correspond to a score that reflects a low chance of selection into discussion. As seen in S1 Table, proposals with scores of 1 are often discussed, and proposals with scores of 5 and above are rarely discussed. Therefore, we map a score of 1 to an “appropriate” decision and a score of 5 to a “gaps identified” decision. When we average these binary decisions across the three reviewers, we get the following scores: 1 (all three reviewers find the proposal “appropriate”), 2.33 (two reviewers select “appropriate” and one selects “gaps identified”), 3.67 (one reviewer selects “appropriate” and two select “gaps identified”), and 5 (all three reviewers select “gaps identified”). Based on the distribution of Investigator and Environment criterion scores (S1 Fig), scores of 1 and 2.33 are more common, while 3.67 and 5 occur less frequently. S1 Table shows that proposals with scores around 1 or 2.33 are more likely to be discussed.

To carry out our thought experiment, we consider two conceptual extremes for how criterion scores relate to Preliminary Overall Impact Scores: the Simultaneous Evaluation Model and the Normatively Backwards Evaluation Model. The Simultaneous Evaluation Model reflects the structure implied by NIH documentation, where all criterion scores are evaluated in parallel and influenced by the underlying PI ability (*U*). Fig 6 shows an example directed acyclic graph for this model. The Normatively Backwards Evaluation Model represents a contrasting scenario in which some criterion scores influence the Preliminary Overall Impact Score while the approach score, $M_{a}$, is evaluated afterward, capturing a potential ordering or dependency in how criteria are assessed. Fig 6 shows an example directed acyclic graph for this model.

**Fig 6:**
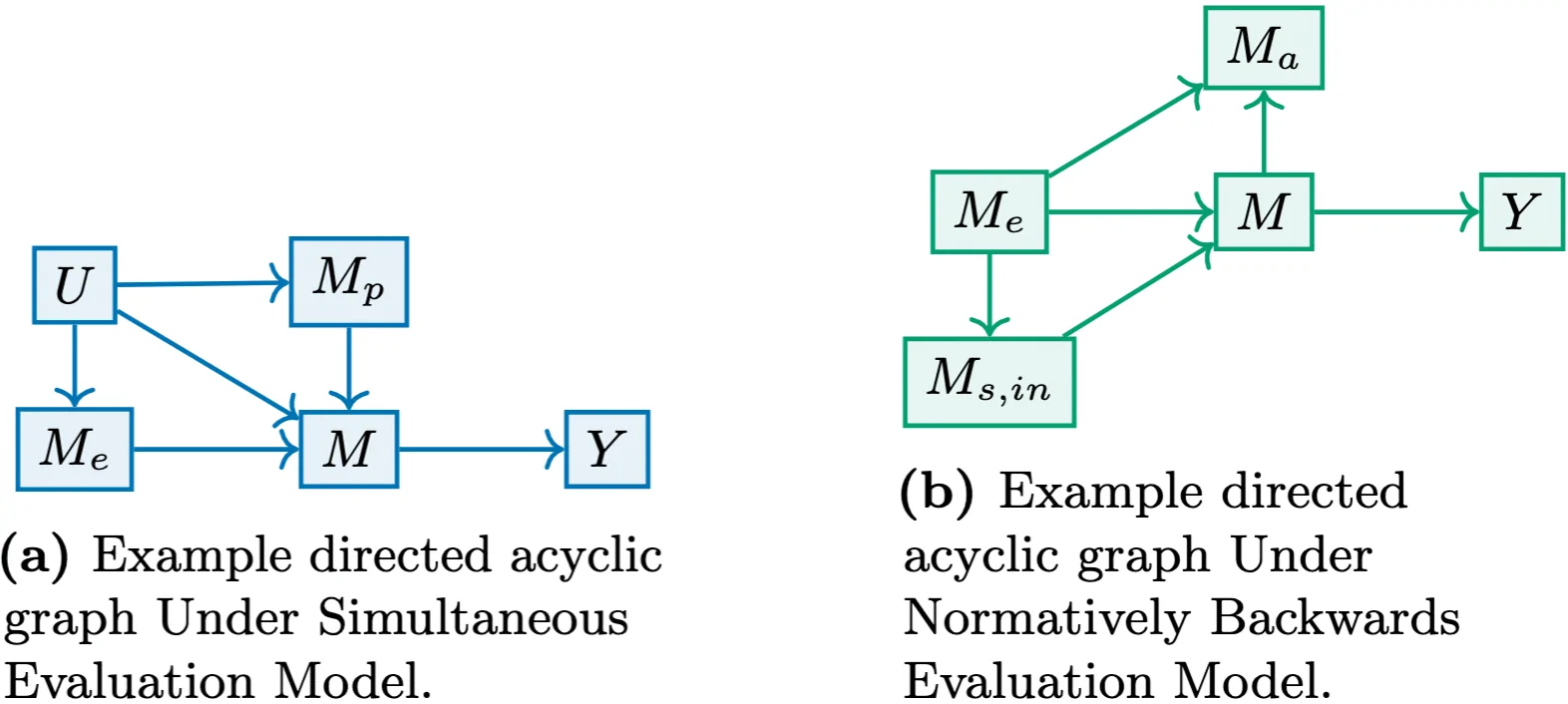
Simplified directed acyclic graphs representing two conceptual extremes for how criterion scores relate to Preliminary Overall Impact Scores. The blue diagram (Simultaneous Evaluation Model) reflects the structure implied by NIH documentation, where all criterion scores are evaluated in parallel and influenced by the underlying PI ability (*U*). The green diagram (Normatively Backwards Evaluation Model) represents a contrasting scenario in which some criterion scores influence the Preliminary Overall Impact Score, and others (notably the approach score, $M_{a}$) are evaluated afterward, capturing a potential ordering or dependency in how criteria are assessed. In both directed acyclic graphs, $M_{e}$ denotes applicant-related criterion scores, $M_{p}$ denotes proposal-related scores, $M_{s,in}$ denotes significance and innovation scores, *M* denotes Preliminary Overall Impact Scores, $M_{a}$ denotes approach scores, and *Y* denotes selection into discussion.

Fig 7 shows results for the Environment criterion score threshold of 2 and Investigator criterion score thresholds from 1 to 3. We focus on this range because higher thresholds are less realistic given historical NIH discussion rates. The combination of an Investigator criterion score threshold of 3 and Environment criterion score threshold of 2 is most plausible, as approximately 50% of proposals with these scores or lower are discussed. We note that thresholded disparities are consistent across Environment criterion score thresholds; therefore, we present results only for the Environment criterion score threshold of 2. Complete results over the full range of threshold combinations are provided in the Supporting Information (see S7 Text and S5 Fig). In what follows, we offer further explanation and intuition for our findings under both the Simultaneous and Normatively Backwards Evaluation Models.

**Fig 7:**
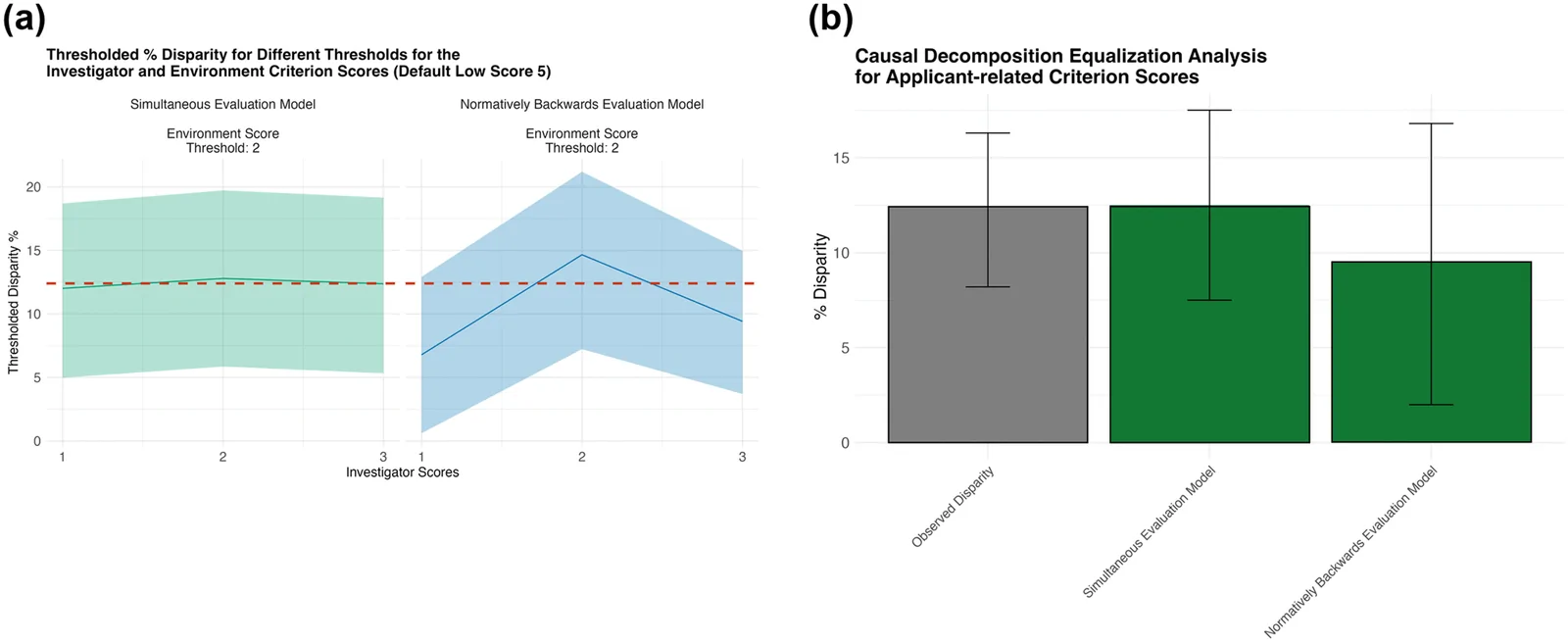
Thresholded disparity and equalization results for applicant-related criterion scores. **(a) Thresholded disparity results.** Each plot shows the percentage of thresholded disparity for an Environment criterion score threshold of 2, with the x-axis representing varying Investigator criterion score thresholds (1-3), based on historical NIH discussion rates. The blue line represents the thresholded disparity under Model 6b, with the blue shaded region indicating the 95% Credible Intervals. The green line represents the thresholded disparity under Model 6a, with the green shaded region indicating the corresponding 95% Credible Intervals. The red dotted line marks the overall observed disparity, which is 12.3% (11.3%, 16.3%). For both models, applications with scores below the Environment and Investigator criterion score thresholds were treated as if they had received a score of 5. **(b) Equalization results.** The plot presents the estimated overall disparity and Disparity Remaining after equalizing applicant-related criterion scores under the Simultaneous and Normatively Backwards Evaluation Models. Positive values indicate disparity against Black PIs. Estimates and their 95% Credible Intervals are obtained using Bayesian estimation.

For the Simultaneous Evaluation Model, we use the estimand identified in Eq S8. Notably, this model conditions on proposal-based criterion scores. Under this model, there is minimal variation across different Investigator and Environment criterion score thresholds because, conditional on the proposal-related criterion scores, the applicant-related criterion scores have minimal impact on selection into discussion, as indicated by the coefficients in the logistic regression model. As a result, regardless of whether reviewers were strict, moderate, or lenient in judging proposals as “acceptable” versus “gaps identified,” disparities in selection into discussion remain largely unchanged from the originally observed levels.

For the Normatively Backwards Evaluation Model, we use the estimand from Eq S9. Notably, this model does not account for proposal-based criterion scores. Our results show that, if reviewers were very strict, deeming only top-tier Investigator scores as “appropriate”, disparities in selection into discussion would become slightly lower than observed, as this strictness would apply to all PIs. Our exploratory analysis supports these results (see S1 Table), as substantial disparities still exist at an Investigator criterion score of 1. If reviewers were to use the Investigator criterion score threshold of 2, meaning that proposals with Investigator criterion scores of 1 or 2 would be considered “appropriate” while scores above 2 would indicate “gaps identified,” this would result in the largest disparities in selection into discussion compared to all other thresholds. This finding can be traced back to data (see S1 Table) that shows that most white PIs receive scores of 1 or 2, while many fewer Black PIs achieve these scores, resulting in the largest Black-white disparity by the Investigator criterion score breakdowns. If reviewers were to use the Investigator criterion score threshold of 3, the disparity in selection into discussion would be similar to that at the threshold of 1. This is because, as shown in S1 Table, the disparity at a score of 3 is the same as that at a score of 1. For higher thresholds (not shown in Fig 7, refer to S5 Fig), disparities decrease slightly and level out for thresholds of 5 and above, as few PIs score above 4. Overall, if reviewers were to binarize Investigator and Environment criterion scores, the expected thresholded disparity differs from the observed disparity, but not substantially, especially for the relevant thresholds of 1–3.

We connect the binarization analysis to the equalization analysis by noting that extremely lenient/strict thresholds effectively treat nearly all Investigator and Environment criterion scores as “acceptable”/“gaps identified” for both Black and white PIs, thereby equalizing their distributions. The findings from our binarization analysis align with those from hypothetical interventions that equalize applicant-related criterion scores, as shown in Fig 7. The identification formulas for these equalization interventions under the Simultaneous and Normatively Backwards Evaluation Models are provided in S6 Table. Under the Simultaneous Evaluation Model, equalization has no impact on disparities – consistent with the binarization analysis results. Under the Normatively Backwards Evaluation Model, equalization leads to small, but not statistically substantial, reductions in disparities, again echoing the binarization analysis results. These results indicate that even under extreme thresholds, where applicant-related scores are effectively equalized across groups, disparities in selection into discussion persist.

Overall, under both the Normatively Backwards and Simultaneous Evaluation models, our thought experiment–-simulating the NIH’s shift to binary Investigator and Environment scores–-did not lead to a meaningful reduction in disparities. The joint evaluation of Investigator and Environment falls into the patterns described in Fig 2. Our conclusions are conditional on the assumed plausible mappings between scoring systems and reviewer behavior, as well as on the assumptions of the causal decomposition framework. Under these conditions, the transition to the Simplified Peer Review Framework is unlikely to substantially reduce disparities.

## Discussion

Following on Hoppe et al.’s [7] finding of substantial Black-white disparity in selection into discussion, we employ a causal decomposition methodology (1) to evaluate what factors drive Black-white disparities in selection for panel discussion and (2) to assess the potential impact of the move to the Simplified Peer Review Framework on these disparities.

In our work, we illustrate that there is quite a bit of variability in both discussion rates and amount of disparity across NIH’s Integrated and Scientific Review Groups. This variability, along with NIH’s documented practices of “rescuing” applications for discussion [13], suggests that selection for discussion is not entirely deterministic based on Preliminary Overall Impact Scores (see S2 Text and S4 Table).

Nonetheless, under the assumptions of the causal decomposition framework, our analysis suggests that eliminating racial disparities in Preliminary Overall Impact Scores can potentially dissolve disparities in selection into discussion. This could be because the selection process is either primarily driven by Overall Impact scores and/or because the variability in discussion selection practices is too infrequent to detect differences. Given the causal influence of Preliminary Overall Impact Scores on selection into discussion, future work should focus on identifying upstream, policy-relevant, and practically feasible interventions for closing disparities in these scores. In the context of NIH grant review, experiments have provided mixed results about the impact of interventions on perceived PI race on Preliminary Overall Impact scores: on the one hand, an experiment that redacted names on proposals submitted by Black, matched white, and random white PIs (400 proposals for each group) found that anonymization halved the difference in mean Preliminary Overall Impact scores for proposals submitted by Black versus white PIs [12]; in contrast, a Goldberg-style study [24] that swapped the perceived-race of applicant names on otherwise identical applications (which included 24 funded and 24 unfunded NIH grant proposals) found little to no difference in assigned Preliminary Overall Impact Scores [18]. Future research is needed to resolve these discrepancies and to evaluate whether inequities in Preliminary Overall Impact Scores could be causally explained by structural inequities that have been found to account for downstream gaps in funding, including inequities in bibliometric profiles [5] and co-author networks [5,8]. Note that research topics disproportionately studied by Black PIs is not listed here because, although such proposals receive less funding, this was found to be due to differences in funding award rates at the Centers and Institutes to which they are assigned rather than differences in peer review scores [6].

Under the assumptions of the causal decomposition framework, our analysis also finds that eliminating disparities in application type, amended status, degree category, institution prestige, career stage, Integrated Review Groups (IRGs) and/or Administering Organization has little potential to reduce disparity in selection into discussion. This finding is akin to that from an earlier correlational analysis that found that preliminary criterion scores fully explained racial disparities in Preliminary Overall Impact Scores [10].

In our thought experiment, the causal decomposition analysis indicates that the impact of moving to binary scoring for the Investigator and Environment criteria in the new Simplified Peer Review Framework depends on how reviewers evaluate the criteria as well as on the underlying causal relationship between the criterion scores and Preliminary Overall Impact Scores. According to our thought experiment, if the process operates as intended in NIH documentation, binarization produces essentially no change in Black–white disparities. Under an alternative, backward evaluation of scores, binarization can have some effect, but the changes supported by the data were not substantial. Under the assumptions of the causal decomposition framework and the scoring scenarios considered in our thought experiment, the results suggest that while efforts to eliminate racial disparities in Preliminary Overall Impact Scores may greatly reduce disparities in selection for discussion, transition to the Simplified Peer Review Framework is unlikely to do so.

Our study has several important limitations. First, due to data privacy restrictions, we did not have access to the full set of applications from 2014 to 2016. Our dataset is only representative of those applications with complete demographic information. We cannot be certain that applicants who did not provide their demographic information were not different from those that did. Second, our thought experiment related only to binarizing the Investigator and Environment criteria scores in the Simplified Peer Review Framework. While it is conceptually straightforward to imagine that reviewers might still assess Investigator and Environment separately before forming a combined binary judgment (see Fig 2), it is not clear how to operationalize the mapping for another important change of combining Significance and Innovation scores into one single Factor 1 score on a 1–9 scale. Third, our analyses relied on patterns in data collected under the previous Enhanced Peer Review framework, and one cannot be certain that data patterns that were present under NIH’s Enhanced Peer Review will persist under the new Simplified Peer Review Framework.

Lastly, while our study includes important applicant- and application-level factors such as career stage, institutional prestige, and whether an application was amended, other potentially influential factors are missing. In particular, we do not have bibliometric profiles [25] or mentorship network measures for applicants [8], which have been found to explain a significant portion of the Black/white R01 funding gap. While we believe the variables we included are sufficient to identify our causal quantities of interest, incorporating additional factors may improve the precision and robustness of our estimates.

This paper demonstrates how a causal decomposition framework could be used to pilot policy initiatives via specifying the updated estimands and identification assumptions and verifying whether those assumptions hold in practice as we did in the second part of the results section. Although the analyses in this paper focused specifically on selection of proposals for panel discussion, our causal decomposition framework could be extended to study hypothetical interventions at other stages such as funding decisions, if the assumptions outlined in the causal decomposition overview section remain reasonable.

Overall, conditional on the assumptions of the causal decomposition framework and on the assumed plausible mappings between scoring systems and reviewer behavior, our findings suggest that interventions such as binarizing the Investigator and Environment criterion scores, as proposed in the Simplified Peer Review Framework, are unlikely to improve Black–white disparities in selection into discussion. We find that, among the proposal and investigator characteristics considered, racial disparities in Preliminary Overall Impact Scores play the central role, suggesting that future work should focus on identifying upstream, policy-relevant, and practically feasible interventions that could reduce disparities in these scores and move selection into discussion toward parity.

## Supporting information

S1 Text Exploratory data analysis. (PDF)

S1 Table Summary statistics for Preliminary Overall Impact, Investigator and Environment Scores. (PNG)

S2 Table Summary statistics for proposal and PI characteristics. Substantial differences and disparities are bolded.
(PNG)

S1 Fig Distributions of Mean Preliminary Overall Impact Scores Conditioned on PI race. (PNG)

S2 Fig Distributions of IRG and Administering Organization Conditioned on PI race. (PNG)

S3 Table Summary statistics for discussion and PI race. (PNG)

S2 Text Selection into discussion and Preliminary Overall Impact Scores. (PDF)

S4 Table Logistic regression model with selection into discussion as the outcome, with PI race, ethnicity, gender, average Preliminary Overall Impact Scores, and other covariates as fixed effects, and SRG included as a random effect. For brevity, we report only those estimates whose 95% credible intervals do not include zero.
(PNG)

S3 Text Materials and Methods. Includes sections S3.1 Data sources, S3.2 Causal decomposition analysis, and S3.3 Estimation.
(PDF)

S5 TableSummarizes the interventions of interest, the estimands of interest, identification assumptions, and identification forms for all interventions considered in the equalization analysis.We use additional notation to the notation introduced in the causal decomposition overview section: PI attributes (career stage, degree category, and institution funding bin, a proxy for institution prestige) as $Z_{pi}$ and proposal attributes (application type and amended status) as $Z_{prop}$.(PNG)

S3 Fig Simplified Interference Structure for two proposals *i* and *j* in a single SRG *k.* (PNG)

S6 TableFor interventions targeting the applicant-related criterion scores, we summarize the estimands of interest, identification assumptions, and identification forms for both the Simultaneous and Normatively Backwards Evaluation Model.We study the effects of both the equalization-based interventions and the thresholding-based interventions on disparities in selection into discussion. In addition to the notation introduced in the causal decomposition overview section, we denote applicant-related criterion scores as $M_{e}$ and proposal-related criterion scores as $M_{p}$.(PNG)

S4 Text Simulation Study. (PDF)

S7 Table Estimator Bias According to Simulations. Use Monte Carlo to generate *D* = 100 datasets of the same data-generating process and calculate the mean bias under the shifted estimator, with and without accounting for interference. Additionally, we compute 95% Credible Intervals. Investigator and Environment criterion score thresholds set to 2.
(PNG)

S5 Text Indentification Proofs. Includes identification proofs of Disparity Reduction and Remaining. Also includes identification of disparity after thresholding scores.
(PDF)

S6 Text Selection. (PDF)

S4 Fig Causal Diagram of NIH Peer Review Process after accounting for Selection Bias. This diagram illustrates the causal relationships within the NIH peer review process. In this representation, *A* corresponds to the PI race, with *A* = 0 representing white PIs and *A* = 1 representing Black PIs. The outcome, *Y*, signifies the selection of proposals for discussion. The selection is denoted as *S*. *W* denotes the PI’s ethnicity and gender, regarded as protected attributes not currently under examination. We allow for an association between ethnicity, gender (*W*), and PI race (*A*), as indicated by the dotted bi-directional arrow. Intermediate variables linking PI race to the selection into discussion include: *M*, the Preliminary Overall score, *Z*, the Application Type, Amended Status, Career Stage, Degree Category, and Funding Bin jointly, and *L*, the Integrated Review Group (IRG), Scientific Review Group (SRG), and Administering Organization.
(PNG)

S7 Text Full Thresholded Disparity Results. (PDF)

S5 Fig Thresholded disparity results. Each plot represents different thresholds for the Environment criterion score for thresholds from 1 to 9. Each plot shows the % Thresholded Disparity for that Environment criterion score threshold and varying Investigator criterion score thresholds for thresholds from 1 to 9 also based on historical NIH discussion rates. The top plot (all in green) corresponds to the Thresholded Disparity under Model 6a. The green shaded region represents the point-wise Credible Intervals corresponding to that model. The bottom plot (all in blue) corresponds to the Thresholded Disparity under Model 6b. The blue shaded region represents the point-wise 95% Credible Intervals corresponding to that model. For both models, scores that fell below the Environment and Investigator criterion score thresholds were treated as if they received an Environment and Investigator criterion score of 5. The red dotted line is the overall observed disparity, which is 12.3% (11.3%, 16.3%).
(PNG)
