# Supplementary material for: Causal decomposition of Black-white disparities in NIH’s selection of proposals for panel discussion
Source: PLoS One. 2026 Oct 28;21(10):e0358838. doi: 10.1371/journal.pone.0358838 (PMC13716228; doi:10.1371/journal.pone.0358838)
Supplement: Supplementary file 1 [file pone.0358838.s001.pdf]

S1 Exploratory Data Analysis

In what follows, we present an exploratory analysis of the distributions of preliminary scores, proposal characteristics, PI characteristics, structural factors, and selection into discussion—focusing on how these distributions differ between Black and white PIs.

Summary Statistics for Preliminary Overall Impact scores Table S1 presents summary statistics related to Preliminary Overall Impact Scores and Investigator and Environment criterion scores.

Summary Statistics for Proposal and PI Characteristics Table S2 presents summary statistics for proposal and PI characteristics.

Distribution of Preliminary Overall Impact scores Figure S1 shows the empirical distributions of Preliminary Overall Impact Scores conditioned on PI race.

Distribution of Structural Characteristics Figure S2 shows the empirical distributions of IRG and Administering Organization conditioned on PI race.

Summary Statistics for Discussion and PI race Table S3 presents summary statistics for discussion and PI race.
