# Supplementary material for: Causal decomposition of Black-white disparities in NIH’s selection of proposals for panel discussion
Source: PLoS One. 2026 Oct 28;21(10):e0358838. doi: 10.1371/journal.pone.0358838 (PMC13716228; doi:10.1371/journal.pone.0358838)
Supplement: Supplementary file 2 [file pone.0358838.s007.pdf]

S2 Selection into Discussion and Preliminary Overall Impact Scores

The analyses in this section indicate that the selection of proposals for panel discussion is not a fully deterministic process with respect to Preliminary Overall Impact Scores. Although our data lack indicators for which applications within each SRG were reviewed together in the same panel meeting, we can still support this claim through two key observations. First, we fit a logistic regression model for selection into discussion (Y), including PI race, ethnicity, gender, average Preliminary Overall Impact Scores, and other applicant, proposal, and structural attributes. Second, as noted in our preliminary analysis, we observe substantial variability in discussion rates and disparities across IRGs and SRGs. Together, these analyses suggest that while these Preliminary Overall Impact scores play a central role, there may be other additional factors that influence which applications are ultimately selected for discussion.

We model selection into discussion (Y) using logistic regression with PI race, ethnicity, gender, average Preliminary Overall Impact Scores, and other covariates as fixed effects, and SRG as a random effect. We compute 95% credible intervals for each coefficient. The analysis reveals that Preliminary Overall Impact Scores have a substantial effect, along with career stage and some individual IRGs (estimates reported in Table S4). This suggests additional contributors to the selection process beyond Preliminary Overall Impact Scores. While the regression analysis suggests that other variables besides Preliminary Overall Impact Score are associated with selection into discussion, it does not allow us to assess their causal contribution to observed Black-white disparities in selection into discussion. To address this, we apply a causal decomposition framework for observational data, extending the approach introduced by VanderWeele et al. [1].

Additionally, our preliminary analysis demonstrated substantial variability in discussion rates and disparities across IRGs and SRGs. This variability provides further evidence that, although Preliminary Overall Impact Scores are the primary driver of selection into discussion, the process does not yield uniform discussion rates across SRGs – suggesting some flexibility at the level of SRGs rather than strict, score-based determination.

Overall, our analysis suggests that selection into panel discussion is primarily driven by Preliminary Overall Impact Scores but that selection is not entirely deterministic.
