# Supplementary material for: Causal decomposition of Black-white disparities in NIH’s selection of proposals for panel discussion
Source: PLoS One. 2026 Oct 28;21(10):e0358838. doi: 10.1371/journal.pone.0358838 (PMC13716228; doi:10.1371/journal.pone.0358838)
Supplement: Supplementary file 3 [file pone.0358838.s009.pdf]

S3 Materials and Methods

S3.1 Data Sources

We analyze publicly available preliminary peer review data on NIH R01 grant applications reviewed by the Center for Scientific Review (CSR) from 2014–2016 [2]. The original dataset contains 4,651 applications with proposal-, investigator-, and review-level information, including PI race, gender, ethnicity, degree type, career stage, institutional funding bin, application type, amended status, Integrated and Scientific Review Group identifiers, administering Institute or Center, and both preliminary and final scoring outcomes. We restructure the data to the proposal level to focus on selection into discussion. Preliminary scores are averaged across reviewers for each proposal, reflecting NIH procedures in which initial decisions are based on average Preliminary Overall Impact Scores [3]. We restrict the sample to applications with three reviewers—the most common configuration—for consistency. The binary outcome indicating selection into discussion is defined based on the availability of a final Overall Impact Score, which is assigned only to proposals that are discussed. This yields a final analytic sample of 3,687 proposals (22% from Black PIs and 78% from white PIs).

S3.2 Causal Decomposition Analysis

In the causal decomposition overview section, we summarize the causal decomposition framework applied to peer review. We demonstrated our estimands of interest: the Observed Disparity, Disparity Reduction, Disparity Remaining, and Thresholded Disparity. To identify these estimands, we account for the interference introduced by the streamlining procedure. Because proposals are reviewed in parallel and relative to one another within study sections, streamlining may result in interventions on one proposal potentially influencing the outcomes of other proposals within the same study section, introducing interference. However, it is reasonable to assume partial interference, meaning that interference occurs only within each SRG [4].

To illustrate the concept of partial direct interference, Ogburn and VanderWeele [5] describe a scenario involving two individuals living in the same household, each randomized to an intervention aimed at lowering cholesterol. The intervention includes cooking classes, nutritional counseling, and coupons for fresh produce. If only one household member receives the intervention, they may still bring fresh produce into the home, prepare healthier meals, or share advice from the nutritionist—indirectly improving the untreated individual's diet. The untreated individual may still experience improvements in blood cholesterol—not through direct intervention, but because exposure to the treated household environment can influence dietary habits and affect cholesterol levels. As this interference occurs only within households, it constitutes an instance of partial direct interference.

We encounter a parallel situation in NIH peer review. Under the streamlining procedure, changes to the score of one proposal can influence which other proposals in the same study section are selected for discussion—resulting in direct interference. As in the cholesterol example, this interference is localized (i.e., partial interference): just as the household limits who is affected, in our setting, interference occurs only within SRGs. This allows us to formalize our framework using an approach similar to that proposed by Ogburn and VanderWeele [5].

Let n denote the total number of proposals and N the number of SRGs. Proposals are partitioned into SRGs indexed by k, with n_k proposals in SRG k. Let $\mathbf{M}_k = (M_{k1}, \ldots, M_{kn_k})$ denote the vector of scores within SRG k. Let $\mathbf{m} = (\mathbf{m}_1, \ldots, \mathbf{m}_N)$ denote a global assignment vector across all SRGs. For proposal i in SRG k, define the potential outcome $Y_{ki}(\mathbf{m}_k)$ as the outcome that would be observed under an intervention that sets $\mathbf{M}_k = \mathbf{m}_k$.

We represent partial interference using the simplified structure depicted in the DAG in Figure S3, which closely follows the direct interference example shown in Figure 4 of [5]. This DAG illustrates a basic setting involving only two proposals, i and j, within a single SRG k. While no SRG in practice evaluates only two proposals, this simplified setting is sufficient to illustrate the type of interference under consideration and the variables that must be adjusted for to control confounding. To simplify the representation, all variables that confound the relationships between M_i and Y_i are grouped into a single node $C_i \equiv (W_i, A_i, Z_i, L_i)$. The DAG implies that adjusting for C_i suffices to block all backdoor paths from both M_i and M_j to Y_i, and thus $Y_i(m_i, m_j) \perp\!\!\!\perp (M_i, M_j) \mid C_i$. This permits identification of the causal effect of the score vector $\mathbf{M}$ on Y_i under partial interference.

In Section S5.1, we present the identification proofs for the Disparity Reduction and Remaining. We estimate the Disparity Reduction and Remaining for a variety of interventions, equalizing the Black and white PIs' distributions of: 1) mean Preliminary Overall Impact Score, 2) application type and amended status jointly, 3) degree category, funding bin, and career stage jointly, and 4) administering organization and IRG jointly. To identify the Disparity Reduction and Disparity Remaining under these interventions we require 1) partial interference, 2) consistency at the SRG level, 3) the effect of the intervention variable on the outcome Y is unconfounded at the SRG level, and 4) positivity at the SRG level. In Table S5, we summarize the interventions, the estimands of interest, and the specific forms of the identification assumptions for the equalization analysis.

We also use our causal decomposition framework to analyze the impact of thresholding Environment and Investigator criterion scores using the Thresholded Disparity. Specfically, we study the Thresholded Disparity under both the Simultaneous

and Normatively Backwards Evaluation Models. Formal identification results for both models are provided in Section S5.2. In our analysis, we connect the binarization analysis to the equalization analysis by noting that extreme thresholds effectively equalize the distributions between Black and white PIs. In Table S6, we summarize the estimands of interest (Disparity Reduction, Disparity Remaining, and Thresholded Disparity), identification assumptions, and identification forms for these interventions on the applicant-related criterion scores under both the Simultaneous and Normatively Backwards Evaluation Models.

S3.3 Estimation

S3.3.1 Estimation Framework

To estimate the Disparity Reduction, Disparity Remaining, and thresholded disparity, we consider only interventions that keep the discussion rate constant. This ensures that all aspects of review other than the distributions of the intervened attributes remain unchanged. Let d_k denote the observed discussion rate for SRG k, and n_k the number of proposals in SRG k. Define

$$Y_k(\cdot) = \frac{1}{n_k}\sum_{i=1}^{n_k} Y_{ki}(\cdot)$$

as the proportion of proposals selected for discussion in SRG k. In order to estimate the Disparity Reduction, Disparity Remaining and thresholded disparity, we must estimate Eqs. (S1) and (S2).

$$\mathbb{P}\big(Y_{ki}(\mathbf{M}_k^t) = 1 \mid A_{ki} = 0\big), \quad \text{s.t.} \quad \mathbb{E}\big[Y_k(\mathbf{M}_k^t)\big] = d_k. \tag{S1}$$

$$\mathbb{P}\big(Y_{ki}(\mathbf{M}_{e,k}^t) = 1 \mid A_{ki} = 0\big), \quad \text{s.t.} \quad \mathbb{E}\big[Y_k(\mathbf{M}_{e,k}^t)\big] = d_k. \tag{S2}$$

Under the identifying assumptions described in the causal decomposition overview, the Disparity Reduction, Disparity Remaining and thresholded disparity can be written as functionals of the observed data distribution. To estimate these functionals under interventions that preserve the observed discussion rate within each SRG, we introduce the *shifted estimator*.

We begin by specifying a working model for the probability of discussion. Let C_{ki} denote a vector of observed covariates for proposal i in SRG k, chosen to capture the variables required for identification, including those that account for interference within SRGs. The specific covariates included in C_{ki} depend on the identification form associated with each estimand (e.g., thresholded disparity versus disparity reduction), but we use a common notation for simplicity. Following [2], we incorporate an SRG-specific random effect and fit a logistic regression model of the form

$$\hat{p}_{k,i} = \frac{1}{1 + \exp(-(\beta_0 + \beta_a A_{ki} + \beta_c^\top C_{ki} + b_k))},$$

where b_k is an SRG-specific random effect.

To enforce the constraint that the discussion rate in each SRG equals the observed rate d_k, we introduce an SRG-specific shift parameter γ_k. The shifted predicted probability is defined as

$$\hat{p}_{k,i}^{\text{shift}} = \frac{1}{1 + \exp(-(\beta_0 + \gamma_k + \beta_a A_{ki} + \beta_c^\top C_{ki} + b_k))}.$$

The shift parameter γ_k is chosen to satisfy

$$\frac{1}{n_k} \sum_{i=1}^{n_k} \hat{p}_{k,i}^{\text{shift}} = d_k, \qquad k = 1, \ldots, N,$$

which ensures that, within each SRG, the average shifted predicted probability matches the observed discussion rate. Intuitively, this estimator enforces the marginal constraint by shifting the intercept, aligning the overall discussion rate with d_k while preserving the relative ordering of proposals within each SRG.

Under the working model, the shifted predicted probabilities $\hat{p}_{k,i}^{\text{shift}}$ approximate the conditional expectation under the model. Averaging over the empirical distribution of the covariates yields a plug-in estimate of the corresponding marginal expectations $\mathbb{E}[Y(\mathbf{M}_e^t) \mid A = a]$ and $\mathbb{E}[Y(\mathbf{M}^t) \mid A = a]$.

Thus, the Disparity Reduction, Disparity Remaining, and thresholded disparity can all be expressed through a common plug-in form:

$$\widehat{\Delta} = \frac{1}{n_{A=0}} \sum_{k=1}^{N} \sum_{i:A_{ki}=0} \hat{p}_{k,i}^{\text{shift}} - \frac{1}{n_{A=1}} \sum_{k=1}^{N} \sum_{i:A_{ki}=1} \hat{p}_{k,i}^{\text{shift}}. \tag{S3}$$

The specific estimand (e.g., Disparity Reduction) is determined by the definition of the shifted probabilities $\hat{p}_{k,i}^{\text{shift}}$, which depend on the corresponding intervention and identification form.

S3.3.2 Bayesian Implementation

We estimate Disparity Reduction, Disparity Remaining and Thesholded Disparity using a Bayesian framework, which provides a coherent way to model the joint distribution of outcomes and mediators, incorporate structural constraints required for the intervention, and propagate uncertainty through the intervention and aggregation steps. Specifically, we combine parametric models for the outcome and mediator distributions with a

group-level Bayesian bootstrap and use Monte Carlo integration to obtain posterior summaries. In order to estimate Disparity Reduction and Disparity Remaining, we follow Algorithm 1; here, we illustrate the procedure by intervening on average Preliminary Overall Impact Scores. In the first step of Algorithm 1, we model selection into discussion Y using a Bernoulli likelihood, with the mean modeled by a logistic regression including fixed effects for the PI's race, ethnicity, gender, and the proposal's average Preliminary Overall Impact Scores, the mean and variance of scores from other proposals within the same SRG, application type, amended status, career stage, degree category, funding bin, IRG, and Administering Organization. SRG is included as a random effect to account for differences across SRGs [2].

Algorithm 1 Bayesian Estimation Procedure for Disparity Reduction and Remaining.

1. Model Y using a Bernoulli likelihood with SRG as a random effect and other attributes as fixed effects.

2. Model M using a Gaussian likelihood with SRG as a random effect.

3. For each proposal i in SRG k:

   (a) Draw 100 samples from $M^*_{ki} \sim P(M_{ki} \mid A_{ki} = 0, W_{ki}, L_{ki})$.

   (b) For each draw, compute summaries $\mu_{M^*_{k,(-i)}}$ and $\sigma^2_{M^*_{k,(-i)}}$ using the sampled intervened scores.

   (c) Estimate

   $$P\Big(Y_{ki}(\mathbf{M}^*_k) = 1 \mid A_{ki}, M_{ki} = M^*_{ki}, \mu_{M^*_{k,(-i)}}, \sigma^2_{M^*_{k,(-i)}}, Z_{ki}, W_{ki}, L_{ki}\Big)$$

   for each sample and average over the Monte Carlo samples.

   - To improve efficiency, we summarize the scores of other proposals within each SRG using $\mu_{M^*_{k,(-i)}}$ and $\sigma^2_{M^*_{k,(-i)}}$, rather than conditioning on each individual score.

4. Apply the shifted estimator to the resulting predicted probabilities so that $E[Y_k(M^*)] = d_k$.

5. Jointly model all other variables using a group-level Bayesian bootstrap and compute $E[Y(M^*) \mid A = 1]$ via posterior averaging.

6. Compute Disparity Reduction and Remaining.

To account for interference, we allow a proposal's discussion outcome to depend on the scores of other proposals within the same SRG, but assume no interference across SRGs. To make estimation tractable, we do not model the full vector of other proposals' scores directly. Instead, conditional on a proposal's own score and observed covariates, we assume that the effect of other proposals' scores on selection into discussion is captured by the mean and variance of those scores within the same SRG, denoted $\mu_{M^*_{k,(-i)}}$ and $\sigma^2_{M^*_{k,(-i)}}$. This working modeling assumption reduces the dimensionality of the problem. Intuitively, we assume $\mu_{M^*_{k,(-i)}}$ and $\sigma^2_{M^*_{k,(-i)}}$ summarize the overall level and dispersion of scores among competing proposals in the same SRG. We assess the adequacy of this approximation (i.e., representing the influence of other proposals' scores using only their mean and variance) in simulation studies described in Appendix S4.

In the second step of Algorithm 1, we model the average Preliminary Overall Impact Scores using a linear mixed-effects model with Gaussian residuals. Specifically, for proposal i in SRG k,

$$M_{ki} \mid A_{ki}, W_{ki}, L_{ki} \sim \mathcal{N}(\mu_{ki}, \sigma^2), \quad \text{with} \quad \mu_{ki} = \beta_0 + \beta_a A_{ki} + \beta_w W_{ki} + \beta_l L_{ki} + b_k,$$

where race, ethnicity, gender, IRG, and Administering Organization are included as fixed effects, and $b_k \sim \mathcal{N}(0, \tau^2)$ is a random effect for SRG that captures between-SRG variability. In the next step of Algorithm 1, we approximate the counterfactual outcomes under the intervention using Monte Carlo integration.

In the third step of Algorithm 1, for each proposal, we use Monte Carlo integration to average over draws of the intervened score $M^*_{ki} \sim P(M_{ki} \mid A_{ki} = 0, W_{ki}, L_{ki})$. For each draw, we compute the corresponding leave-one-out summaries $\mu_{M^*_{k,(-i)}}$ and $\sigma^2_{M^*_{k,(-i)}}$, and input these leave-one-out summaries into the logistic regression model to estimate $P\Big(Y_{ki}(\mathbf{M}^*_k) = 1 \mid A_{ki}, M_{ki} = M^*_{ki}, \mu_{M^*_{k,(-i)}}, \sigma^2_{M^*_{k,(-i)}}, Z_{ki}, W_{ki}, L_{ki}\Big)$. Averaging over the Monte Carlo samples yields proposal-level predicted probabilities. We then adjust these probabilities using the shifted estimator so that $E[Y_k(M^*)] = d_k$. In the final steps of Algorithm 1, we model the joint distribution of W, L, and Z using a group-level Bayesian bootstrap at the SRG level, and compute $E[Y(M^*) \mid A = 1]$ via posterior averaging. We repeat the model fitting, intervention, and prediction steps for 1,000 draws, obtaining 1,000 estimates of the Disparity Reduction and Disparity Remaining, which are used to construct 95% credible intervals.

In Algorithm 2, we estimate the thresholded disparity using a procedure analogous to that described above for Disparity Reduction and Disparity Remaining (Algorithm 1). As described when introducing the thresholded disparity in Eq. (4), this estimand requires binarizing the Investigator and Environment criterion scores relative to a threshold. We therefore consider thresholds over the support of each score, applied separately to the Investigator and Environment criteria, to indicate whether each score

exceeds the threshold. This allows us to assess how the Thresholded Disparities vary across different cutoff levels. In the first step of Algorithm 2, for each pair of thresholds $(t_{\text{invest}}, t_{\text{envir}})$, selection into discussion Y is modeled using a Bernoulli likelihood, with the mean specified via logistic regression. To define the intervention at a given threshold pair, we threshold the Investigator and Environment criterion scores as:

$$M^t_{e,ki} = \begin{cases} 1, & M^{(\text{invest})}_{e,ki} \leq t_{\text{invest}} \text{ and } M^{(\text{envir})}_{e,ki} \leq t_{\text{envir}}, \\ 5, & \text{otherwise.} \end{cases} \tag{S4}$$

In the next step of Algorithm 2, for each SRG, we apply the thresholding defined in Eq. (S4) to all proposals and use the resulting thresholded scores to compute summaries of the other proposals. Specifically, to obtain predicted probabilities of selection under the intervention, we include $M^t_{e,ki}$ along with the leave-one-out mean and variance of the thresholded scores of other proposals, denoted $\mu_{M^t_{e,k,(-i)}}$ and $\sigma^2_{M^t_{e,k,(-i)}}$, among the covariates in the logistic regression model (with the full set of covariates under the Normatively Backwards and Simultaneous Evaluation models specified in Algorithm 2). The summaries $\mu_{M^t_{e,k,(-i)}}$ and $\sigma^2_{M^t_{e,k,(-i)}}$ capture within-SRG interference. To enforce the fixed discussion rate d_k, we use the shifted estimator. In the final steps of Algorithm 2, we model the remaining variables jointly via a group-level Bayesian bootstrap and compute $E[Y(M^t_e) \mid A = 0]$ and $E[Y(M^t_e) \mid A = 1]$ to estimate the thresholded disparity. We carry out the model fitting, intervention, and prediction steps for 1,000 draws, obtaining 1,000 estimates of the thresholded disparity, which are used to construct 95% credible intervals.

Algorithm 2 Bayesian Estimation Procedure for thresholded disparity.

1. For each pair of thresholds $(t_{\text{invest}}, t_{\text{envir}})$, where $t_{\text{invest}}, t_{\text{envir}} \in \{1, \ldots, 9\}$:

   (a) Model selection into discussion Y using logistic regression with SRG as a random effect and other attributes as fixed effects:

   - For the Normatively Backwards Evaluation Model, model

     $$Y_{ki} \mid A_{ki}, M_{e,ki}, \mu_{M_{e,k,(-i)}}, \sigma^2_{M_{e,k,(-i)}}, W_{ki}, L_{ki}, Z_{ki}.$$

   - For the Simultaneous Evaluation Model, model

     $$Y_{ki} \mid A_{ki}, M_{e,ki}, \mu_{M_{e,k,(-i)}}, \sigma^2_{M_{e,k,(-i)}}, M_{p,ki}, W_{ki}, L_{ki}, Z_{ki}.$$

   (b) Define the thresholded Investigator and Environment criterion scores by

$$M^t_{e,ki} = \begin{cases} 1, & M^{(\text{invest})}_{e,ki} \le t_{\text{invest}} \text{ and } M^{(\text{envir})}_{e,ki} \le t_{\text{envir}}, \\ 5, & \text{otherwise.} \end{cases}$$

(c) Compute summaries $\mu_{M^t_{e,k,(-i)}}$ and $\sigma^2_{M^t_{e,k,(-i)}}$, and use them together with $M^t_{e,ki}$ to obtain predicted probabilities of selection under the intervention:

- For the Normatively Backwards Evaluation Model,

$$P\Big(Y_{ki}(\mathbf{M}^t_{e,k}) = 1 \mid A_{ki}, \mathbf{Z}_k, \mathbf{W}_k, \mathbf{L}_k, M^t_{e,ki}, \mu_{M^t_{e,k,(-i)}}, \sigma^2_{M^t_{e,k,(-i)}}\Big).$$

- For the Simultaneous Evaluation Model,

$$P\Big(Y_{ki}(\mathbf{M}^t_{e,k}) = 1 \mid A_{ki}, \mathbf{Z}_k, \mathbf{M}_{p,k}, \mathbf{W}_k, \mathbf{L}_k, M^t_{e,ki}, \mu_{M^t_{e,k,(-i)}}, \sigma^2_{M^t_{e,k,(-i)}}\Big).$$

(d) Apply the shifted estimator so that $E[Y_k(M^t_e)] = d_k$.

(e) Jointly model the remaining variables using a group-level Bayesian bootstrap and compute $E[Y(M^t_e) \mid A = 0]$ and $E[Y(M^t_e) \mid A = 1]$ by posterior averaging.

(f) Compute the thresholded disparity.

In our Bayesian estimation framework (illustrated in Algorithms 1 and 2), we use weakly informative priors because the data are sufficiently informative for the likelihood to dominate [6]. Fixed-effect parameters in the logistic regression have $N(0, 4)$ priors, and SRG random effects follow $N(\mu_{srg_y}, \sigma_{srg_y})$ with $\mu_{srg_y} \sim N(0, 4)$ and $\sigma_{srg_y} \sim \text{Exponential}(1)$. For the linear regression modeling average scores, fixed-effect parameters have $N(0, 10)$ priors, with analogous priors for SRG random effects.

Predictive posterior and MCMC diagnostic checks reveal no issues.

Moreover, in our Bayesian estimation framework, summaries such as $\mu_{M_{k,(-i)}}$ and $\sigma^2_{M_{k,(-i)}}$ are, by construction, highly correlated within SRGs. However, because our target of inference is the marginal distribution $P(Y \mid A)$, rather than conditional relationships involving these summaries, such within-group dependence has limited impact on inference. To further account for this dependence, we implement a group-level Bayesian bootstrap, assigning resampling weights at the SRG level [7,8]. Results closely match those from the standard Bayesian bootstrap, suggesting that within-group dependence does not substantially affect our estimates.
