# Supplementary material for: Causal decomposition of Black-white disparities in NIH’s selection of proposals for panel discussion
Source: PLoS One. 2026 Oct 28;21(10):e0358838. doi: 10.1371/journal.pone.0358838 (PMC13716228; doi:10.1371/journal.pone.0358838)
Supplement: Supplementary file 4 [file pone.0358838.s013.pdf]

S4 Simulation Study

The goal of our simulation study is to assess the statistical bias of the shifted estimator under the true data-generating process and to evaluate the impact of interference. To do this, we simulate data under a set of simplifying assumptions.

To simplify the hierarchical structure of the NIH review process, we assume that SRGs, IRGs, and Administering Organizations are fully nested (i.e., $SRG \rightarrow IRG \rightarrow Admin$). Each SRG is treated as an independent unit and corresponds to a single study section meeting, with 100 proposals per SRG – consistent with typical NIH study section sizes, which range from 60 to 100 proposals [9].

For each SRG, we simulate proposal-level data based on a structural equation model (SEM) that encodes the assumptions of our causal diagrams. Specifically, we first sample PI ethnicity and gender (W) and PI race (A) independently with replacement from their empirical marginal distributions for the matched SRG. Then, conditional on W and A, we draw additional application-level attributes - Application Type, Amended Status, Career Stage, Degree Category, and Funding Bin (jointly denoted as Z) - from their empirical conditional distribution. Preliminary criterion scores are then generated using the SEM based on these covariates. This framework assumes conditional independence of proposals within each SRG and independence across SRGs.

We estimate SEM parameters using the matched NIH dataset, which exhibits fewer extreme class imbalances. This improved balance enables more robust estimation and reduces sensitivity to model misspecification [2].

This setup allows us to ground our simulations in real NIH data while maintaining a fully known data-generating process. It allows us to assess the statistical bias of our estimates, including bias introduced by interference, under specific interventions, while keeping the simulated data aligned with the structure of the observed data. For simplicity, we assume that selection into discussion is fully deterministic–specifically, the top half of Preliminary Overall Impact Scores in the same study section are selected for discussion.

We present our simulations for the Thresholded Disparity estimand. Since the shifted estimator behaves similarly across all estimands, studying its performance under this estimand provides insight into its overall performance across our estimands of interest. Through our simulations, we aim to quantify the overall bias in our estimates, which includes the bias arising from interference. To do so, we must first define the *truth*. In our simulation setting, the truth is the true disparity in selection into discussion after jointly binarizing Investigator and Environment criterion scores. This specification explicitly accounts for the fact that exactly 50% of proposals are selected based solely on Preliminary Overall Impact Scores.

We use linear mixed models with SRG as a random effect to learn the structural equation model parameters from the NIH matched dataset:

- $M_e|A, W, L, Z$
- $M_i|A, W, L, Z$
- $M_s|A, W, L, Z, M_e, M_i$
- $M_{inn}|A, W, L, Z, M_e, M_i$
- $M|A, W, L, Z, M_e, M_i, M_s, M_{inn}$
- $M_a|A, W, L, Z, M_e, M_i, M$

Using these models, we simulate data following Algorithm 3. For each Preliminary Overall Impact Score, we introduce noise based on within-SRG variability estimated from the linear mixture models. We then compute the true Thresholded Disparity using the known data-generating process (Algorithm 4).

Algorithm 3 Simulating Data

for admin org **in the** Admin Orgs **do**
  for irg **in** admin org **do**
    for srg **in** srg **do**

1. Compute $P(W|SRG = srg)$ and $P(A|SRG = srg)$ using NIH matched data
2. Simulate 100 proposals for this SRG:
   - $SRG = srg, IRG = irg$, Admin Org = admin org
   - Sample W with replacement using $P(W|SRG = srg)$
   - Sample A with replacement using $P(A|SRG = srg)$
   - Sample Z with replacement using $P(Z|SRG = srg, A = a, W = w)$
   - Using linear mixed models, predict:
     - $M_{\text{environment}}$ adding noise $\eta_{m_e} \sim N(0, \sigma^2_{m_e})$, where $\sigma^2_{m_e}$ is the estimated within-SRG variability
     - $M_{\text{investigator}}$ adding noise $\eta_{m_i} \sim N(0, \sigma^2_{m_i})$, where $\sigma^2_{m_i}$ is the estimated within-SRG variability
     - $M_{\text{significance}}$ adding noise $\eta_{m_s} \sim N(0, \sigma^2_{m_s})$, where $\sigma^2_{m_s}$ is the estimated within-SRG variability
     - $M_{\text{innovation}}$ adding noise $\eta_{m_{inn}} \sim N(0, \sigma^2_{m_{inn}})$, where $\sigma^2_{m_{inn}}$ is the estimated within-SRG variability
     - M adding noise $\eta_m \sim N(0, \sigma^2_m)$, where σ^2_m is the estimated within-SRG variability
     - M_{approach} adding noise $\eta_{m_a} \sim N(0, \sigma^2_{m_a})$, where $\sigma^2_{m_a}$ is the estimated within-SRG variability

- Rank proposals based on M and set selection into discussion Y as 1 for the top 50% of proposals and 0 otherwise

end for
end for
end for

Algorithm 4 Using Simulation to Get True Thresholded Disparity

for admin org **in** Admin Orgs **do**
  for irg **in** admin org **do**
    for srg **in** srg **do**

1. Get the simulated data for $SRG = srg$
2. For every proposal in this SRG, threshold $M_{i,e}$ using $M^t_{i,e} = \begin{cases} 1, M^{new}_{i,e} \leq \begin{bmatrix} 2 \\ 2 \end{bmatrix} \\ 5, M^{new}_{i,e} > \begin{bmatrix} 2 \\ 2 \end{bmatrix} \end{cases}$
3. Using linear mixed models, obtain:
   - $M_s(M^t_{i,e})|A, W, L, Z, M^t_{i,e}$, adding noise $\eta_{m_s} \sim N(0, \sigma^2_{m_s})$, where $\sigma^2_{m_s}$ is the estimated within-SRG variability
   - $M_{inn}(M^t_{i,e})|A, W, L, Z, M^t_{i,e}$, adding noise $\eta_{m_{inn}} \sim N(0, \sigma^2_{m_{inn}})$, where $\sigma^2_{m_{inn}}$ is the estimated within-SRG variability
   - $M(M^t_{i,e})|A, W, L, Z, M^t_{i,e}, M_s(M^t_{i,e}), M_{inn}(M^t_e)$, adding noise $\eta_m \sim N(0, \sigma^2_m)$, where σ^2_m is the estimated within-SRG variability
4. Rank proposals based on $M(M^t_{i,e})$ and set selection into discussion after the intervention, $Y(M^t_{i,e})$, as 1 for the top 50% of proposals and 0 otherwise

    end for
  end for
end for
Use empirical mean to estimate $E[Y(M^t_e) = 1|A, M_e = m^t_e, Z, W, L]$ and estimate Thresholded Disparity

---

We use Monte Carlo to generate $D = 100$ datasets of the same data-generating process and calculate the bias under our shifted estimator, with and without accounting for interference. We set the Investigator and Environment criterion score thresholds at 2.

Table S7 reports the average estimator bias in the Thresholded Disparity, both with and without accounting for interference. In both cases, the bias is small, and the shifted estimator is approximately unbiased when interference is accounted for. Overall, the simulation results suggest that interference has minimal effect on our estimates.
