# Supplementary material for: Causal decomposition of Black-white disparities in NIH’s selection of proposals for panel discussion
Source: PLoS One. 2026 Oct 28;21(10):e0358838. doi: 10.1371/journal.pone.0358838 (PMC13716228; doi:10.1371/journal.pone.0358838)
Supplement: Supplementary file 5 [file pone.0358838.s015.pdf]

S5 Identification Proofs

We prove identification for Disparity Reduction, Disparity Remaining, and Thresholded Disparity estimands when there is no selection bias.

S5.1 Identification Proofs of Disparity Reduction and Remaining

We identify Disparity Reduction and remaining for the case of equalizing Preliminary Overall Impact Scores. This identification proof holds for the other interventions we study and the differences in the unconfounding and positivity assumptions and the identification forms given in Table S5.

$$
\begin{aligned}
P(Y_{ki}(\mathbf{M}_k = \mathbf{M}_k^*)|A_{ki}=1,\mathbf{w}_k,\mathbf{l}_k) &= \sum_{\mathbf{m}_k} P(Y_{ki}(\mathbf{M}_k=\mathbf{m}_k)|A_{ki}=1,\mathbf{w}_k,\mathbf{l}_k,\mathbf{M}_k^*=\mathbf{m}_k) \\
&\quad \times P(\mathbf{M}_k^*=\mathbf{m}_k|A_{ki}=1,\mathbf{w}_k,\mathbf{l}_k) \quad \text{(LOTP \& consistency)} \\
&= \sum_{\mathbf{m}_k} P(Y_{ki}(\mathbf{M}_k=\mathbf{m}_k)|A_{ki}=1,\mathbf{w}_k,\mathbf{l}_k) \\
&\quad \times P(\mathbf{M}_k^*=\mathbf{m}_k|A_{ki}=1,\mathbf{w}_k,\mathbf{l}_k) \\
&\text{since } Y_{ki}(\mathbf{M}_k=\mathbf{m}_k) \perp \mathbf{M}_k^*=\mathbf{m}_k|\{\mathbf{A}_k,\mathbf{W}_k,\mathbf{L}_k\} \text{ by definition of} \\
&\mathbf{M}_k^* \text{ as a random draw given } \mathbf{W}=w_k, \mathbf{L}_k=l_k \\
&= \sum_{\mathbf{m}_k} P(Y_{ki}(\mathbf{M}_k=\mathbf{m}_k)|A_{ki}=1,\mathbf{w}_k,\mathbf{l}_k) \\
&\quad \times P(\mathbf{M}_k^*=\mathbf{m}_k|A_{ki}=0,\mathbf{w}_k,\mathbf{l}_k) \\
&\quad \text{We have } \mathbf{M}_k^*=\mathbf{m}_k^* \perp \mathbf{A}_k|\mathbf{W}_k,\mathbf{L}_k \text{ by definition of } \mathbf{M}_k^* \text{ as a} \\
&\text{random draw given } \mathbf{W}_k,\mathbf{L}_k \\
&= \sum_{\mathbf{m}_k} P(Y_{ki}(\mathbf{M}_k=\mathbf{m}_k)|A_{ki}=1,\mathbf{w}_k,\mathbf{l}_k) \\
&\quad \times P(\mathbf{M}_k=\mathbf{m}_k|A_{ki}=0,\mathbf{w}_k,\mathbf{l}_k) \\
&\quad \text{definition of } \mathbf{M}_k^* \sim P(\mathbf{M}_k|A_{ki}=0,\mathbf{W}_k,\mathbf{L}_k) \\
&= \sum_{\mathbf{m}_k,\mathbf{z}_k} P(Y_{ki}(\mathbf{M}_k=\mathbf{m}_k)|A_{ki}=1,\mathbf{w}_k,\mathbf{l}_k,\mathbf{z}_k) \\
&\quad \times P(\mathbf{z}_k|A_{ki}=1,\mathbf{w}_k,\mathbf{l}_k) \\
&\quad \times P(\mathbf{M}_k=\mathbf{m}_k|A_{ki}=0,\mathbf{w}_k,\mathbf{l}_k) \quad \text{(LOTP)} \\
&= \sum_{\mathbf{m}_k,\mathbf{z}_k} P(Y_{ki}(\mathbf{M}_k=\mathbf{m}_k)|A_{ki}=1,\mathbf{w}_k,\mathbf{l}_k,\mathbf{m}_k,\mathbf{z}_k) \\
&\quad \times P(\mathbf{z}_k|A_{ki}=1,\mathbf{w}_k,\mathbf{l}_k) \\
&\quad \times P(\mathbf{M}_k=\mathbf{m}_k|A_{ki}=0,\mathbf{w}_k,\mathbf{l}_k), \text{ since } (\mathbf{Y}(\mathbf{m}_k) \perp \mathbf{M}_k|\mathbf{A}_k,\mathbf{W}_k,\mathbf{L}_k,\mathbf{Z}_k) \\
&\sum_{\mathbf{m}_k,\mathbf{z}_k} P(Y_{ki}|A_{ki}=1,\mathbf{w}_k,\mathbf{l}_k,\mathbf{m}_k,\mathbf{z}_k)P(\mathbf{z}_k|A_{ki}=1,\mathbf{w}_k,\mathbf{l}_k) \\
&\quad \times P(\mathbf{M}_k=\mathbf{m}_k|A_{ki}=0,\mathbf{w}_k,\mathbf{l}_k) \quad \text{(consistency)}
\end{aligned}
$$

Let $M = \sum_{k=1}^{N} n_k$.

$$
\begin{aligned}
E[Y(\mathbf{M}^*)|A=1] &= \frac{1}{M}\sum_{k=1}^{N}\sum_{i=1}^{n_k}\sum_{\mathbf{w}_k,\mathbf{l}_k,\mathbf{m}_k,\mathbf{z}_k} P(Y_{ki}(\mathbf{M}^*)|A_{ki}=1,\mathbf{w}_k,\mathbf{l}_k)P(\mathbf{w}_k,\mathbf{l}_k|A_{ki}=1) \\
&= \frac{1}{M}\sum_{k=1}^{N}\sum_{i=1}^{n_k}\sum_{\mathbf{w}_k,\mathbf{l}_k,\mathbf{m}_k,\mathbf{z}_k} P(Y_{ki}|A_{ki}=1,\mathbf{w}_k,\mathbf{l}_k,\mathbf{m}_k,\mathbf{z}_k) \\
&\quad \times P(\mathbf{z}_k,\mathbf{w}_k,\mathbf{l}_k|A_{ki}=1)P(\mathbf{M}_k=\mathbf{m}_k|A_{ki}=0,\mathbf{w}_k,\mathbf{l}_k)
\end{aligned}
$$

We can apply this derivation to multiple mediators. When there are multiple mediators we can model the mediators as follows:

$$
\begin{aligned}
P(\mathbf{m}=(m_1,m_2,\cdots,m_k)|w,l,A=0) &= P(m_1|w,l,A=0) \\
&\quad \times P(m_2|m_1,w,l,A=0) \\
&\quad \times \cdots P(m_k|m_1,\cdots,m_{k-1},w,l,A=0)
\end{aligned}
$$

S5.2 Identification of Disparity after Thresholding Scores

The identification would be very similar to the identification for Disparity Reduction and remaining (Section S5.1). We prove identification under the Normatively Backwards Evaluation Model, but similar steps can be made to get the identification form under the Simultaneous Evaluation Model.

$$
\begin{aligned}
P(Y_{ki}(\mathbf{M}_{e,k}=\mathbf{M}^t_{e,k})|\mathbf{a}_k,\mathbf{w}_k,\mathbf{l}_k,\mathbf{z}_k) &= \sum_{\mathbf{m}_{e,k}\in\{m^t\}} P(Y_{ki}(\mathbf{M}_{e,k}=\mathbf{m}_{e,k})|\mathbf{a}_k,\mathbf{w}_k,\mathbf{l}_k,\mathbf{z}_k,\mathbf{M}^t_{e,k}=\mathbf{m}_{e,k}) \\
&\quad \times P(\mathbf{M}^t_{e,k}=\mathbf{m}_{e,k}|\mathbf{a}_k,\mathbf{w}_k,\mathbf{l}_k,\mathbf{z}_k) \quad \text{(LOTP \& consistency)} \\
&= \sum_{\mathbf{m}_{e,k}\in\{m^t\}} P(Y_{ki}(\mathbf{M}_{e,k}=\mathbf{m}_{e,k})|\mathbf{a}_k,\mathbf{w}_k,\mathbf{l}_k,\mathbf{z}_k) \\
&\quad \times P(\mathbf{M}^t_{e,k}=\mathbf{m}_{e,k}|\mathbf{a}_k,\mathbf{w}_k,\mathbf{l}_k,\mathbf{z}_k) \\
&\quad Y_{ki}(\mathbf{M}_{e,k}=\mathbf{m}_{e,k}) \perp \mathbf{M}^t_{e,k}=\mathbf{m}_{e,k}|\{\mathbf{A}_k,\mathbf{W}_k,\mathbf{L}_k,\mathbf{Z}_k\} \text{ since} \\
&\quad Y_{ki}(\mathbf{M}_{e,k}=\mathbf{m}_{e,k}) \perp \mathbf{M}^e_k=\mathbf{m}_{e,k} \mid \{\mathbf{A}_k,\mathbf{W}_k,\mathbf{L}_k,\mathbf{Z}_k\}) \\
&\quad \text{and we fix } \mathbf{M}^t_{e,k}=\mathbf{M}_{e,k}=\mathbf{m}_{e,k} \\
&= \sum_{\mathbf{m}_{e,k}\in\{m^t\}} P(Y_{ki}(\mathbf{M}_{e,k}=\mathbf{m}_{e,k})|\mathbf{a}_k,\mathbf{w}_k,\mathbf{l}_k,\mathbf{z}_k,\mathbf{M}_{e,k}=\mathbf{m}_{e,k}) \\
&\quad \times P(\mathbf{M}^t_{e,k}=\mathbf{m}_{e,k}|\mathbf{a}_k,\mathbf{w}_k,\mathbf{l}_k,\mathbf{z}_k) \\
&\quad \text{(by independence: } Y_{ki}(\mathbf{m}_{e,k}) \perp \mathbf{M}_{e,k} \mid \{\mathbf{A}_k,\mathbf{W}_k,\mathbf{L}_k,\mathbf{Z}_k\}) \\
&= \sum_{\mathbf{m}_{e,k}\in\{m^t\}} P(Y_{ki}|\mathbf{a}_k,\mathbf{w}_k,\mathbf{l}_k,\mathbf{z}_k,\mathbf{M}_{e,k}=\mathbf{m}_{e,k}) \\
&\quad \times P(\mathbf{M}^t_{e,k}=\mathbf{m}_{e,k}|\mathbf{a}_k,\mathbf{w}_k,\mathbf{l}_k,\mathbf{z}_k) \quad \text{(consistency)}
\end{aligned}
$$

Let $M = \sum_{k=1}^{N} n_k$. Then we have that under the Normatively Backwards Evaluation Model:

$$E_{CD}[Y(\mathbf{M}^{e,t})|A=1] = \frac{1}{M}\sum_{k=1}^{N}\sum_{i=1}^{n_k}\sum_{\mathbf{w}_k,\mathbf{l}_k,\mathbf{z}_k} E[Y_{ki}(\mathbf{M}_k^e = \mathbf{M}_k^{e,t})|\mathbf{w}_k,\mathbf{l}_k,\mathbf{z}_k,\mathbf{A}_k=1]P(\mathbf{w}_k,\mathbf{l}_k,\mathbf{z}_k|\mathbf{A}_k=1)$$
$$= \frac{1}{M}\sum_{k=1}^{N}\sum_{i=1}^{n_k}\sum_{\mathbf{w}_k,\mathbf{l}_k,\mathbf{z}_k,\mathbf{m}_k^e\in\{m^t\}} E[Y_{ki}|\mathbf{w}_k,\mathbf{l}_k,\mathbf{z}_k,\mathbf{A}_k=1,\mathbf{M}_k^e=\mathbf{m}_k^e]$$
$$\times P(\mathbf{M}_k^{e,t}=\mathbf{m}_k^e|\mathbf{w}_k,\mathbf{l}_k,\mathbf{z}_k,\mathbf{A}_k=1)P(\mathbf{w}_k,\mathbf{l}_k,\mathbf{z}_k|\mathbf{A}_k=1) \quad \text{(S8)}$$

Similarly, the identification form for the Thresholded Disparity under the Simultaneous Evaluation Model is:

$$E_{Doc}[Y(\mathbf{M}^{e,t})|A=1] = \frac{1}{M}\sum_{k=1}^{N}\sum_{i=1}^{n_k}\sum_{\mathbf{w}_k,\mathbf{l}_k,\mathbf{z}_k,\mathbf{m}_{p,k}} E[Y_{ki}(\mathbf{M}_k^e = \mathbf{M}_k^{e,t})|\mathbf{w}_k,\mathbf{l}_k,\mathbf{m}_{p,k},\mathbf{z}_k,\mathbf{A}_k=1]$$
$$\times P(\mathbf{w}_k,\mathbf{l}_k,\mathbf{z}_k,\mathbf{m}_{p,k}|\mathbf{A}_k=1)$$
$$= \frac{1}{M}\sum_{k=1}^{N}\sum_{i=1}^{n_k}\sum_{\mathbf{w}_k,\mathbf{l}_k,\mathbf{z}_k,\mathbf{m}_{p,k},\mathbf{m}_k^e\in\{m^t\}} E[Y_{ki}|\mathbf{w}_k,\mathbf{l}_k,\mathbf{z}_k,\mathbf{m}_{p,k},\mathbf{A}_k=1,\mathbf{M}_k^e=\mathbf{m}_k^e]$$
$$\times P(\mathbf{M}_k^{e,t}=\mathbf{m}_k^e|\mathbf{w}_k,\mathbf{l}_k,\mathbf{z}_k,\mathbf{m}_{p,k},\mathbf{A}_k=1)P(\mathbf{w}_k,\mathbf{l}_k,\mathbf{z}_k,\mathbf{m}_{p,k}|\mathbf{A}_k=1) \quad \text{(S9)}$$
