# Supplementary material for: Causal decomposition of Black-white disparities in NIH’s selection of proposals for panel discussion
Source: PLoS One. 2026 Oct 28;21(10):e0358838. doi: 10.1371/journal.pone.0358838 (PMC13716228; doi:10.1371/journal.pone.0358838)
Supplement: Supplementary file 6 [file pone.0358838.s016.pdf]

S6 Selection

We define self-selection as the process of applicants' choice to apply for the NIH grant in the first place (denoted as $S = 1$). Figure S4, shows our DAG after accounting for this selection. In this setting, we are interested in the following estimands:

- Observed Disparity: $E[Y|A = 1, S = 1] - E[Y|A = 0, S = 1]$
- Thresholded Disparity:
  $E[Y(M^t)|A = 0, S = 1] - E[Y(M^t)|A = 1, S = 1] \quad s.t \quad P(Y_k(M^t) = 1|S = 1) = d_k$
- Disparity Reduction:
  $E[Y|A = 1, S = 1] - E[Y(M^*)|A = 1, S = 1] \quad s.t \quad P(Y_k(M^*) = 1|S = 1) = d_k$
- Disparity Remaining:
  $E[Y(M^*)|A = 1, S = 1] - E[Y|A = 0, S = 1] \quad s.t \quad P(Y_k(M^*) = 1|S = 1) = d_k$

Where $M^* \sim P(M|A = 0, S = 1, W, L)$ and M^t has the same definition as in the causal decomposition overview section. We are interested in these quantities for the selected population or the population that applied for NIH grants. The identification assumptions and proof are identical to what was shown previously in the causal decomposition overview section, S5.1 and S5.2 with the extra conditioning on $S = 1$.

So the final identification forms are the following:

$$E[Y(\mathbf{M}^*)|A = 1, S = 1] = \frac{1}{M}\sum_{k=1}^{N}\sum_{i=1}^{n_k}\sum_{\mathbf{w}_k,\mathbf{l}_k,\mathbf{m}_k,\mathbf{z}_k,S_{ki}=1} P(Y_{ki}|A_{ki} = 1, \mathbf{w}_k, \mathbf{l}_k, \mathbf{m}_k, \mathbf{z}_k, S_{ki} = 1)$$
$$\times P(\mathbf{z}_k, \mathbf{w}_k, \mathbf{l}_k|A_{ki} = 1, S_{ki} = 1)P(\mathbf{M}_k = \mathbf{m}_k|A_{ki} = 0, \mathbf{w}_k, \mathbf{l}_k, S_{ki} = 1)$$

$$E_{CD}[Y(\mathbf{M}^{e,t})|A = 1, S = 1] = \frac{1}{M}\sum_{k=1}^{N}\sum_{i=1}^{n_k}\sum_{\mathbf{w}_k,\mathbf{l}_k,\mathbf{z}_k,\mathbf{m}_k^e\in\{m^t\}} E[Y_{ki}|\mathbf{w}_k, \mathbf{l}_k, \mathbf{z}_k, A_{ki} = 1, \mathbf{M}_k^e = \mathbf{m}_k^e, S_{ki} = 1]$$
$$\times P(\mathbf{M}_k^{e,t} = \mathbf{m}_k^e|\mathbf{w}_k, \mathbf{l}_k, \mathbf{z}_k, A_{ki} = 1, S_{ki} = 1)P(\mathbf{w}_k, \mathbf{l}_k, \mathbf{z}_k|A_{ki} = 1, S_{ki} = 1)$$

$$E_{Doc}[Y(\mathbf{M}^{e,t})|A = 1, S = 1] = \frac{1}{M}\sum_{k=1}^{N}\sum_{i=1}^{n_k}\sum_{\mathbf{w}_k,\mathbf{l}_k,\mathbf{z}_k,\mathbf{m}_{p,k},\mathbf{m}_k^e\in\{m^t\}}$$
$$E[Y_{ki}|\mathbf{w}_k, \mathbf{l}_k, \mathbf{z}_k, \mathbf{m}_{p,k}, A_{ki} = 1, \mathbf{M}_k^e = \mathbf{m}_k^e, S_{ki} = 1]$$
$$\times P(\mathbf{M}_k^{e,t} = \mathbf{m}_k^e|\mathbf{w}_k, \mathbf{l}_k, \mathbf{z}_k, \mathbf{m}_{p,k}, A_{ki} = 1, S_{ki} = 1)$$
$$\times P(\mathbf{w}_k, \mathbf{l}_k, \mathbf{z}_k, \mathbf{m}_{p,k}|A_{ki} = 1, S_{ki} = 1)$$
