# Supplementary material for: Causal decomposition of Black-white disparities in NIH’s selection of proposals for panel discussion
Source: PLoS One. 2026 Oct 28;21(10):e0358838. doi: 10.1371/journal.pone.0358838 (PMC13716228; doi:10.1371/journal.pone.0358838)
Supplement: Supplementary file 7 [file pone.0358838.s018.pdf]

S7 Full Thresholded Disparity Results

Figure S5 shows the results for all binarization thresholds. In the Normatively Backwards Evaluation Model, thresholds of 4 or higher substantially reduce disparities. However, as noted earlier, such high thresholds may be unlikely given historical NIH discussion rates. In the Simultaneous Evaluation Model, binarizing the Investigator and Environment criterion scores has no impact on disparities, regardless of the threshold.
